# Divergence in *cis-*regulatory sequences surrounding the opsin gene arrays of African cichlid fishes

**DOI:** 10.1186/1471-2148-11-120

**Published:** 2011-05-09

**Authors:** Kelly E O'Quin, Daniel Smith, Zan Naseer, Jane Schulte, Samuel D Engel, Yong-Hwee E Loh, J Todd Streelman, Jeffrey L Boore, Karen L Carleton

**Affiliations:** 1 Department of Biology, University of Maryland, College Park, MD 20742, USA; 2 School of Biology, Petit Institute for Bioengineering and Bioscience, Georgia Institute of Technology, Atlanta, GA 30332 USA; 3 Genome Project Solutions, Hercules, CA 94547, USA; 4 Department of Integrative Biology, University of California, Berkeley, CA 94720, USA

## Abstract

**Background:**

Divergence within *cis*-regulatory sequences may contribute to the adaptive evolution of gene expression, but functional alleles in these regions are difficult to identify without abundant genomic resources. Among African cichlid fishes, the differential expression of seven opsin genes has produced adaptive differences in visual sensitivity. Quantitative genetic analysis suggests that *cis*-regulatory alleles near the *SWS2*-*LWS *opsins may contribute to this variation. Here, we sequence BACs containing the opsin genes of two cichlids, *Oreochromis niloticus *and *Metriaclima zebra*. We use phylogenetic footprinting and shadowing to examine divergence in conserved non-coding elements, promoter sequences, and 3'-UTRs surrounding each opsin in search of candidate *cis*-regulatory sequences that influence cichlid opsin expression.

**Results:**

We identified 20 conserved non-coding elements surrounding the opsins of cichlids and other teleosts, including one known enhancer and a retinal microRNA. Most conserved elements contained computationally-predicted binding sites that correspond to transcription factors that function in vertebrate opsin expression; *O. niloticus *and *M. zebra *were significantly divergent in two of these. Similarly, we found a large number of relevant transcription factor binding sites within each opsin's proximal promoter, and identified five opsins that were considerably divergent in both expression and the number of transcription factor binding sites shared between *O. niloticus *and *M. zebra*. We also found several microRNA target sites within the 3'-UTR of each opsin, including two 3'-UTRs that differ significantly between *O. niloticus *and *M. zebra*. Finally, we examined interspecific divergence among 18 phenotypically diverse cichlids from Lake Malawi for one conserved non-coding element, two 3'-UTRs, and five opsin proximal promoters. We found that all regions were highly conserved with some evidence of CRX transcription factor binding site turnover. We also found three SNPs within two opsin promoters and one non-coding element that had weak association with cichlid opsin expression.

**Conclusions:**

This study is the first to systematically search the opsins of cichlids for putative *cis-*regulatory sequences. Although many putative regulatory regions are highly conserved across a large number of phenotypically diverse cichlids, we found at least nine divergent sequences that could contribute to opsin expression differences in *cis *and stand out as candidates for future functional analyses.

## Background

Adaptive phenotypic evolution may result either from protein-coding mutations that modify the structure and function of genes, or from regulatory mutations that alter the timing, location, or expression of genes [1-3]. Although examples of protein-coding mutations that contribute to phenotypic evolution are well known (e.g., [4-6]), examples of regulatory mutations that also affect phenotypic adaptation are less well known, but no less important (e.g., [7-9]). One class of regulatory mutations, *cis*-regulatory mutations, are found in close proximity to the genes they regulate and function by altering the binding of transcription factors necessary for gene expression. *Cis*-regulatory mutations exhibit several features that make them ideally suited for adaptive phenotypic evolution, including codominance [10] and modularity [8]. These features make *cis*-regulatory mutations efficient targets for natural selection [11] and limit the negative consequences of pleiotropy that presumably affect many *trans*-regulatory and protein-coding mutations. Finally, since *cis*-regulatory mutations may underlie many of the adaptive and disease phenotypes found in nature, identifying these alleles remains an important goal of evolutionary genetics. However, identifying *cis*-regulatory mutations can be challenging without abundant functional genomic resources, since the transcription factor binding sites (TFBS) they affect are small, lack strict conservation, and are found in difficult-to-annotate regions of the genome [2,3].

The location of *cis*-regulatory sequences can be near-to or far-from the genes they regulate. Promoter sequences found directly upstream of genes can harbor *cis*-regulatory alleles [12,13], as can enhancer or repressor elements located many kilobases away [14,15]. *Cis*-regulatory sequences can even reside within the untranslated regions (UTRs) of genes, where they alter the binding of microRNAs (miRNAs) that regulate gene expression following transcription [16,17]. But where ever their location, two methods commonly used to identify *cis*-regulatory sequences and alleles are phylogenetic footprinting and phylogenetic shadowing [18]. In phylogenetic footprinting, one compares DNA surrounding some gene(s) of interest among numerous divergent taxa in hopes of identifying non-coding regions that are highly conserved. By the very nature of their conservation, these conserved non-coding elements (CNEs) stand out as candidate regulatory sequences, since conservation is often used to indicate function. Once candidate regulatory sequences have been identified via phylogenetic footprinting, the method used to identify putative *cis*-regulatory alleles within them is differential phylogenetic footprinting, or phylogenetic shadowing [18,19]. In phylogenetic shadowing, one compares putative regulatory sequences among closely related taxa in hopes of identifying sequence polymorphisms correlated with the divergent expression of some target gene(s). Following their application, functional genomic analyses are necessary to validate the function of any candidate sequences or alleles identified by the phylogenetic footprinting and shadowing methods; but even by themselves, both methods can provide valuable insights into the location of potential *cis*-regulatory sequences and the transcription factors that bind them.

**Figure 1:**
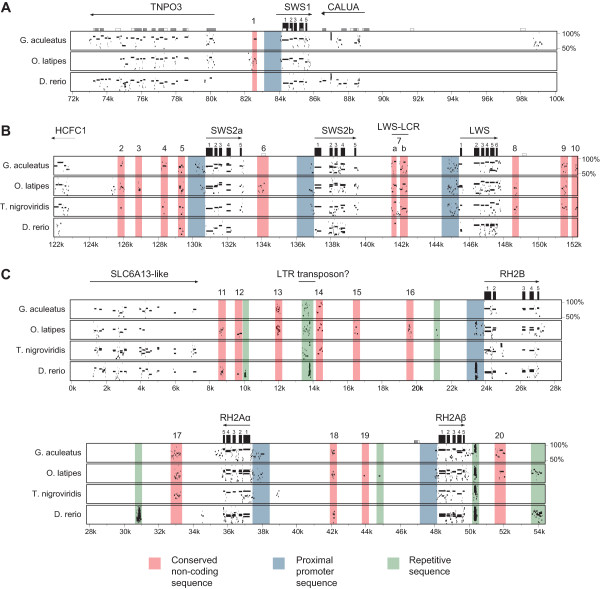
**Conservation between *O. niloticus *opsin-containing BAC regions and four fish genomes**. A) *SWS1 *opsin-containing region. B) *SWS2-LWS *opsin-containing region. C) *RH2 *opsin-containing region. Top line represents *O. niloticus *BAC sequence. Conserved non-coding elements (CNEs) are numbered and highlighted in red; repetitive sequences are highlighted in green; promoter sequences later examined for interspecific polymorphism are highlighted in blue.

The goal of this study is to identify candidate *cis*-regulatory sequences that control opsin gene expression in African cichlid fishes. Opsins are a group of G protein-coupled receptors that confer sensitivity to light and mediate color vision [20]. African cichlids comprise a diverse clade of freshwater, teleost fish found throughout the lakes and rivers of Africa, including the three African Great Lakes, Lakes Tanganyika, Malawi, and Victoria [21,22]. Cichlids from Lakes Tanganyika and Malawi exhibit dramatic variation in their sensitivity to colored light [23-25]. Species from these lakes exhibit retinal sensitivities that are maximally sensitive to short, middle, or long-wavelength spectra; in some cases, closely related species can differ in their maximal retinal sensitivity by over 100 nm [25-27]. This striking variation makes the cichlid visual system one of the most diverse vertebrate visual systems so far identified. Most variation in cichlid color sensitivity is due to changes in the regulation of their cone opsin genes [26,27]. Cichlids have seven cone opsin genes used for color vision; these opsins are *SWS1 *(ultraviolet-sensitive), *SWS2B *(violet-sensitive), *SWS2A *(blue-sensitive), *RH2B *(blue-green-sensitive), *RH2A *and *RH2A *(green-sensitive), and *LWS *(red-sensitive) [28]. Additionally, these opsins are located in three regions of the cichlid genome: *SWS1 *is found on cichlid linkage group (LG) 17; *RH2B, RH2A *and *RH2A *are found together in a tandem array on LG 5; and *SWS2A, SWS2B*, and *LWS *form a second tandem array on LG 5 (Lee et al. 2005) (Figure 1). Among different cichlid species, these opsins are alternatively co-expressed in three predominant groups, or palettes, to produce the three common visual pigment sets: *SWS1*-*RH2B*-*RH2A *(short wavelength-sensitive), *SWS2B*-*RH2B*-*RH2A *(middle wavelength-sensitive), and *SWS2A*-*RH2A*-*LWS *(long wavelength-sensitive) [26]. Cichlids exhibit several correlations between the expression of their opsins and important ecological variables, including foraging preference and ambient light intensity [26,27]. These correlations suggest that opsin gene expression varies adaptively in cichlids, especially since some expression-ecology correlations have evolved independently among cichlids in different lakes [27]. A recent quantitative genetic analysis of opsin expression in two Lake Malawi cichlids found a quantitative trait locus (QTL) located near the opsin genes [29]. The proximity of this QTL to the opsins suggests that mutations within one or more *cis*-regulatory sequences may contribute to variation in cichlid opsin expression. But like many non-model systems, few genomic resources are currently available for cichlids, making it difficult to identify potential *cis*-regulatory alleles and test their association with opsin gene expression.

Here, we sequence and analyze bacterial artificial chromosome (BAC) clones containing the opsin genes of two African cichlid species, *Oreochromis niloticus *[30] and *Metriaclima zebra *[31]. *Oreochromis niloticus *(the Nile tilapia) is a riverine cichlid that expresses the long wavelength-sensitive opsin palette as adults but also expresses the other palettes as fry and juveniles [32]. *O. niloticus *is an outgroup to the diverse haplochromine cichlids endemic to Lakes Tanganyika, Malawi, and Victoria. *Metriaclima zebra *(the 'classic' Zebra cichlid) is one such haplochromine cichlid found in Lake Malawi. *M. zebra *expresses the short wavelength-sensitive opsin palette as an adult and during all developmental stages [32]. Both species last shared a common ancestor ~ 18 MYA, whereas *M. zebra *diverged from other phenotypically diverse Lake Malawi cichlids less than 2 MYA [33]. After sequencing the opsin-containing BAC clones from these species, we used the resulting sequences for several analyses, including:

(1) Annotation and comparison of the opsin-containing regions from the genome assemblies of several model teleosts. We perform phylogenetic footprinting by comparing the opsin-containing regions of *O. niloticus *and several model fish genomes. We use this comparison to locate conserved non-coding elements (CNEs) that serve as candidate *cis*-regulatory sequences for the opsins.

(2) Computational prediction of binding sites for 12 transcription factors important for vertebrate opsin expression [34-41] (Table 1). We perform this search in each CNE as well as within the proximal promoter of each opsin. We also perform an analogous search for miRNA target sites within the 3'-UTR of each opsin.

**Table 1:** List of candidate transcription factors surveyed in this study

Transcription Factor	Symbol	OMIM^1 ^#	TESS^2 ^# (mice)	Opsin(s) affected	Ref(s)
Activator Protein 1	AP-1	165160	T00032	*SWS1*	[37]
Cone-rod homeobox-protein	CRX/OTX	602225	T03461	*SWS2*	[41]
Nuclear Factor kappa B	NFκB	164011	T00588	*SWS1*	[37]
Photoreceptor-specific nuclear receptor	PNR	604485	T03723*	SWS	[39]
Retinoic Acid Receptor α	RARα	180240	T01327	*SWS1*	[35]
Retinoic Acid Receptor β	RARβ	180220	T01328	*SWS1*	[35]
Retinoic Acid Receptor γ	RARγ	180190	T01329	*SWS1*	[35]
Retinoid X Receptor α	RXRα	180245	T01331	-	-
Retinoid X Receptor β	RXRβ	180246	T01332	-	-
Retinoid X Receptor γ	RXRγ	180247	T01333	SWS	[40]
Thyroid Hormone Receptor α	THRα	190120	T01173	*SWS1*	[36]
Thyroid Hormone Receptor β	THRβ	190160	T00851*	*SWS1, RH2*	[36,38]

^1 ^Online Mendelian Inheritance in Man (http://www.ncbi.nlm.nih.gov/omim)

^2 ^Transcription Element Search System (http://www.cbil.upenn.edu/cgi-bin/tess/tess)

* TESS # for human sequences

(3) Phylogenetic shadowing between *O. niloticus *and *M. zebra *using the TFBS and miRNA target site profiles found in each CNE, promoter, and 3'-UTR sequence. In each region we compare the proportion of divergent TFBS/miRNA target sites with the amount expected given the over-all sequence divergence of the opsin BACs and introns (a measure of neutral evolutionary divergence [42,43]). These comparisons are used to identify putative *cis*-regulatory sequences that have undergone significant evolutionary divergence among African cichlids.

(4) Following phylogenetic shadowing, we re-sequence the most divergent regions in a panel of 18 phenotypically diverse cichlids from Lake Malawi. We search these sequences for polymorphisms that may indicate the presence of *cis*-regulatory alleles. This final analysis allows us to determine whether the divergent regions we identify between *O. niloticus *and *M. zebra *also contain polymorphisms correlated with opsin expression in the more closely related cichlids of Lake Malawi.

We use the final results of this study to examine which regulatory regions are most likely to contain functional regulatory alleles that determine opsin expression in African cichlids. We find that many non-coding regions are highly conserved between *O. niloticus *and *M. zebra*, as well as among the closely related cichlids of Lake Malawi. However, we find at least two CNEs, five proximal promoters, and two 3'-UTRs that exhibit significant divergence in the number and type of TFBS and miRNA targets found between *O. niloticus *and *M. zebra*. We also identify at least three alleles that are weakly associated with *SWS2A, RH2B*, and *LWS *expression - three opsins that show strong differential expression among cichlid species. These results suggest that *cis*-regulatory sequences may contribute to opsin expression differences among African cichlids, and provide numerous candidates for future functional studies.

## Results and Discussion

### BAC Sequencing and Analysis

#### BAC identification, sequencing, assembly, and comparison

Within the cichlid genome, the opsins are found in three separate tandem arrays. *SWS1 *is found alone on cichlid linkage group (LG) 17; *SWS2A, SWS2B*, and *LWS *are found together in a tandem array on LG 5 [44]; and *RH2B, RH2Aα*, and *RH2Aβ *are found in a second tandem array on LG 5 approximately 30 cM from the *SWS2-LWS *array (KL Carleton, unpublished data) [44]. We identified opsin-containing BAC clones for *O. niloticus *by PCR screening [30] and for *M. zebra *by filter hybridization [31]. We then shotgun sequenced each clone using ABI Sanger or 454 Life Sciences technology. Clone IDs, estimated sizes, sequencing methods, assembly statistics, final contig length, and GenBank accession numbers for resulting contigs are listed in Table 2. The average read length for ABI-generated sequences was ~700 bp, while the average read length for 454-generated sequences was ~110 bp. For the *O. niloticus SWS1*-containing clone, we used a combination of ABI and 454 sequences since the assemblies based on ABI-generated reads alone were poor. For all other clones, we used additional Sanger reads to fill in the gaps and join all contigs into their final BAC assemblies (Table 2). Overall, the final assemblies of each clone based on ABI and 454 technology joined an average of 85% of reads into a single contig that was within 10 - 40 kb of the estimated clone size (Table 2). All assemblies successfully covered the opsin-containing regions in *O. niloticus *and *M. zebra*.

**Table 2:** Assembly statistics for the *O. niloticus *and *M. zebra *opsin-containing BACs

Species	Opsin array	Clone ID	Estimated clone size (bp)	Sequencing method	Contig size (bp)	Reads assembled (%)	GenBank accession nos.
*O. niloticus*	*SWS1*	T4057DH09	210,000	ABI, 454	171,838	77 K + 3 K (95 + 49)	JF262087
	*SWS2-LWS*	T4075AE05	184,000	ABI	171,742	3072 (85.1)	JF262088
	*RH2A-RH2B*	T4024BG04	200,000	ABI	177,366	3072 (84.2)	JF262086
*M. zebra*	*SWS1*	Mz042C6	87,000	454	77,652	79,892 (95.2)	JF262085
	*SWS2-LWS*	Mz045P9	96,000	454	107,624	43,135 (93.8)	JF262084
	*RH2A-RH2B*	Mz088M22	133,000	454	83,463	21,758 (94.8)	JF262089

^1 ^Estimated clone size based on Pulsed Gel Electrophoresis.

We aligned each BAC assembly from *O. niloticus *and *M. zebra *and found them to be highly similar. The only significant difference was a 6.1 kb insertion in the *M. zebra RH2*-containing BAC, located between the *RH2Aα*, and *RH2Aβ *opsins (Additional file 1). This insertion is likely a transposon. The average pairwise Jukes-Cantor-corrected sequence divergence (D_xy_) across each BAC assembly was 8.4% (± 3.1% s.e.). This rate of sequence divergence is consistent with comparisons of other genes between these species, and it is one of the first large-scale estimates of sequence divergence between *O. niloticus *and *M. zebra*. We then subdivided each BAC assembly into opsin protein-coding (CDS) and intronic (INT) sequences. For *O. niloticus *and *M. zebra*, the mean D_xy _across all opsin CDS was 3.8% (± 0.3%), while the divergence across all INT was 9.5% (± 1.9%). (We excluded both the first intron as well as the first and last six bases of each intron since these regions may contain regulatory sequences and splice sites that are more highly conserved than other intronic regions [43]). Comparison of the average D_xy _across all regions reveals that the mean divergence of the functionally important opsin CDS is significantly lower than D_xy _across either the BACs or INT sequences (t-tests: CDS vs. BAC, t_8, 0.05 _= 2.60, p = 0.032; CDS vs. INT, t_27, 0.05 _= 2.17, p = 0.039), but that D_xy _between BAC and INT sequences do not differ (t_23, 0.05 _= 0.08, p = 0.935). In addition to evaluating which regions of each opsin-containing BAC retain the highest conservation and are most likely to be functional, these divergence estimates also provide an important null hypothesis for our subsequent analyses using phylogenetic shadowing: in general, we expect *O. niloticus *and *M. zebra *to share (e.g, exhibit orthology in) ~92% of their TFBS and miRNA target sites, and exhibit divergence in ~8%. Divergence in greater than 8% of the TFBS and miRNA target sites identified may indicate significant *cis*-regulatory sequence evolution in the regions examined.

#### BAC annotation and the opsin repertoire of teleost fishes

In order to perform phylogenetic footprinting across the opsin arrays of cichlids, we first investigated the synteny of each opsin array of *O. niloticus *relative to several model fish species using PipMaker [45] and MultiPipMaker [46]. We found considerable synteny in the opsin-containing regions among *O. niloticus *(tilapia), *Gasterosteus aculeatus *(stickleback), *Oryzias latipes *(medaka), *Tetraodon nigroviridis *(tetraodon), and *Danio rerio *(zebrafish) (Figure 1; Additional file 2A). The clearest example of this synteny was the *SWS2*-*LWS *opsin array. This array is flanked by the genes *HCFC1 *and *GNL3L *and is essentially co-linear in all five fish genomes (Figure 1; see Additional file 3 for the position and orientation of flanking genes). We found evidence for a localized duplication of the *SWS2 *opsins in *O. latipes *and *O. niloticus*, since both these species have two adjacent *SWS2 *opsin genes (Additional file 4). Closely related Poeciliid fishes also possess adjacent *SWS2 *paralogs [47], suggesting that this duplication event probably occurred at least 153 - 113 MYA at the base of the Acanthopterygii [48,49].

In contrast to the *SWS2-LWS *array, we observed considerable variation in opsin gene content for the *RH2 *opsins. *O. niloticus *and *M. zebra *possess three *RH2 *genes while *D. rerio *has four [50,51], *G. aculeatus *has two, and *T. nigrovirdis *has one functional *RH2 *opsin and one *RH2 *pseudogene [52]. We therefore used phylogenetic analyses to investigate the orthology of the *RH2 *and *SWS2 *genes among these fishes and found that most *RH2 *duplications are species-specific [53] (Additional File 4). Thus, synteny in the region containing the *RH2 *opsin array was lower than in the *SWS2-LWS *array, but was still largely co-linear between *O. niloticus, G. aculeatus*, and *T. nigroviridis *(Additional file 2B). The genes *SLC6A13-like *and *SYNPR *flank the *RH2 *opsins in these fishes (Figure 1; Additional file 3).

Synteny in the region surrounding the *SWS1 *opsin was difficult to assess due to species-specific deletions and poor genome assembly. The *T. nigrovirdis *genome assembly lacks the *SWS1 *opsin altogether, and this region is found within an unordered chromosome or ultracontig in both the *G. aculeatus *and *O. latipes *genomes. For *G. aculeatus*, we found a small 92 kb region containing the *SWS1 *opsin that was collinear with the *O. niloticus *BAC sequence, but which contained one large inversion. For *O. latipes*, we found an even smaller 60 kb region that was syntenic for only 11 kb surrounding the *SWS1 *opsin. Synteny with *D. rerio *was also generally low (Additional file 2C). Therefore, despite the lack of *SWS1 *duplicates compared to the *SWS2 *or *RH2 *opsins, the *SWS1 *region is still poorly assembled in the existing annotations of several teleost genomes, potentially complicating direct comparisons of synteny in this region. In these species, the *SWS1 *opsin appears to be flanked by the genes *TNPO3 *and *CALUA *(Figure 1; Additional file 3).

### Analysis of Conserved Non-Coding Elements (CNEs)

#### Phylogenetic footprinting to identify CNEs

We used MultiPipMaker [46] to highlight non-coding elements surrounding each opsin gene array from *O. niloticus *to *D. rerio*, representing nearly 300 MY of fish evolution [49]. The resulting plots illustrate at least 20 conserved non-coding elements (CNEs) surrounding the opsin gene arrays of *O. niloticus *and the other fish species examined (red bars in Figure 1). We also found six regions of putatively high conservation that are largely composed of repetitive sequence (green bars in Figure 1), which we did not analyze further. The conservation of these CNEs over several million years of fish evolution suggests that they contain functionally important regulatory modules necessary for gene expression.

**Figure 2:**
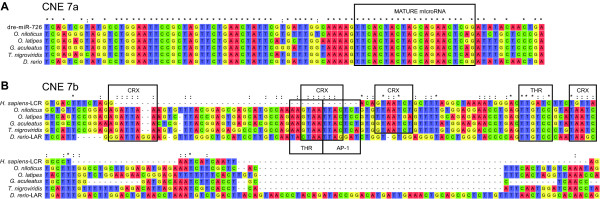
**Alignment of two putative *LWS *opsin regulatory elements (CNE 7a and b) in fishes**. A) Alignment of CNE 7a from five fish genomes to dre-miR-726. This region is highly similar among all fish species examined. Black box indicates the mature microRNA sequence. B) Alignment of CNE 7b from five fish genomes to the human LWS-LCR. These sequences show regions of high similarity between humans and fishes. Asterisks (*) indicate positions that are identical among all taxa; colons (:) indicate positions that are identical among four out of five taxa. Boxes highlight conserved transcription factor binding sites.

At least one CNE we identified through phylogenetic footprinting is orthologous to other vertebrate *cis*-regulatory sequences. CNE 7 (highlighted in Figure 1 and located between the *SWS2B *and *LWS *opsins) consists of two non-contiguous regions of high conservation in pufferfish, stickleback, medaka, swordtails, and cichlids [47] (Figure 1). The first region, CNE 7a, was also identified following a comparative analysis of opsin-containing BACs from swordtails (*Xiphophorus helleri*) [47]. Through BLAST and mirbase [54], we found that CNE 7a is most similar to zebrafish miRNA dre-miR-726 (score 173.3, e-value = 0.006), and the same genomic region from zebrafish is identical to this miRNA (Figure 2). Dre-miR-726 is expressed in the retina of larval and adult zebrafish [55]. Since many miRNAs are transcribed along with the genes they regulate, the proximity of miR-726 to the *SWS2 *and *LWS *opsins suggests that it could play a role in opsin regulation. The ~90 bp CNE encoding mir-726 is conserved in numerous other taxa as well, including additional fishes, frogs, and lizards [47,56].

The second highly conserved region, CNE 7b, is positionally and structurally orthologous to the mammalian *LWS *locus control region (LWS-LCR; Figure 2B) [47,56,57]. This enhancer is located ~3.8 kb upstream of the *LWS *opsin in *O. niloticus *and other vertebrates, including humans. The LWS-LCR is hypothesized to enhance *LWS *expression in eutherian mammals by looping and binding to the *LWS *proximal promoter [57-59]. Wang et al. [59] demonstrated that the human ortholog of this sequence can function as an enhancer of both *LWS *and *MWS *opsin expression in mice. Additionally, a recent study of *LWS *regulation in zebrafish also identified a similar sequence at this position that modulates *LWS *expression in that species, which they named the *LWS *activating region (LAR) [60]. Comparison of the mammalian LWS-LCR, the zebrafish LAR, and CNE 7b from cichlids and other teleosts reveals a high degree of sequence similarity among these regions (Figure 2B). In Figure 2B, we also highlight several conserved transcription factor binding sites common to each sequence, including sites for CRX, THR, and AP-1 (Figure 2B; see also Table 1). Thus, our results demonstrate the effectiveness of the phylogenetic footprinting method for identifying functional *cis*-regulatory sequences necessary for vertebrate opsin expression. It is therefore possible that the remainder of the CNEs we identify also encode *cis*-regulatory sequences necessary for the correct spatial and developmental expression of the opsins in cichlids.

We note that our present study focuses on small regions of high conservation within a ~30 kb window of non-coding sequence surrounding the opsin arrays, but that *cis*-regulatory sequences may often reside tens or hundreds of kilobases from the genes they regulate. However, two recent analyses of general transcription factor binding sites found that functional binding sites generally cluster in regions 1 kb around the proximal promoter of each gene [61,62]. This observation suggests that a focused study of conserved elements within or near the opsins is a reasonable strategy for this initial study. A FASTA file of all CNE sequences from *O. niloticus *and *M. zebra *is provided in Additional file 5.

#### TFBS search and phylogenetic shadowing of CNEs

We compared the 20 CNEs identified between *O. niloticus *and *M. zebra *and found many to be highly conserved; however, we found no identifiable orthologs between *O. niloticus *and *M. zebra *for CNEs 6 or 19. For the remaining CNEs, the average pairwise sequence divergence between *O. niloticus *and *M. zebra *was 4.2% (± 0.5%), which is significantly less than the mean D_xy _of introns (9.5%, t-test: t_38, 0.05 _= 2.99, p = 0.005). This result suggests that the conserved non-coding regions identified among *O. niloticus *and other fishes have remained conserved among African cichlids as well.

We used the Transcription Element Search System [63] to computationally search all orthologous CNEs for binding sites corresponding to twelve transcription factors that have been associated with opsin expression in fishes and other vertebrates including thyroid hormone and retinoic acid receptors [34-37,39,41,64,65]. A complete list of these transcription factors and their associated opsins is presented in Table 1. We found computationally-predicted binding sites for these functionally important transcription factors in all but one of the CNEs surveyed (Table 3; see Additional file 6 for detailed counts of all TFBS). Only CNE 10 lacked binding sites for any of the twelve transcription factors in either species examined. Within the remaining sequences we found binding sites for all twelve transcription factors except PNR and RXRγ. After relaxing our matching criteria, we still failed to find binding sites for these two transcription factors (data not shown). In both *O. niloticus *and *M. zebra*, binding sites for AP-1 and CRX were extremely abundant, although binding sites for each of three retinoic acid receptors (RARs) and THRβ were also common (Additional file 6). We found several CNEs with a high density of transcription factor binding sites given the total sequence length surveyed - generally 9 TFBS or more (see Additional file 6). For *O. niloticus *these high-density CNEs are CNEs 2, 3, 13, 15, 19, and 20, and for *M. zebra *these are CNEs 2, 8, 11, 13, 15, and 20. Due to their potential enrichment for functional TFBSs relative to other CNEs, we believe these eight CNEs represent the most likely candidates for functional *cis*-regulators of opsin expression in fishes.

**Table 3:** Comparison of sequence similarity and TFBS/miRNA target site divergence for putative *cis*-regulatory regions surrounding the opsin arrays of *O. niloticus *and *M. zebra*

Region		Identity	D_xy_^1^	Length	Length	TFBS	TFBS	Est.	p-value^3^
		(%)	(%)	*On *(bp)	*Mz *(bp)	Divt.	Shrd	P_div_^2 ^(%)	
CNE^4^	1	96.84	3.23	158	158	0	2	0.0	1.000
	2	96.22	3.88	240	239	2	6	25.0	0.130
	3	94.74	4.53	349	359	7	1	87.5	< 0.001*
	4	98.31	1.70	240	241	2	0	100.0	0.006
	5	96.14	3.97	207	207	1	0	100.0	0.080
	6	-	-	300	-	-	-	-	-
	7	97.16	2.89	882	885	1	8	11.1	0.528
	8	88.46	4.86	779	799	3	9	25.0	0.065
	9	93.93	6.33	313	313	1	3	25.0	0.283
	10	97.64	2.40	127	127	0	0	-	-
	11	95.97	4.14	124	124	1	1	50.0	0.154
	12	95.53	4.61	246	249	1	3	25.0	0.284
	13	97.66	2.37	214	214	1	9	10.0	0.566
	14	88.97	4.71	999	1404	1	9	10.0	0.566
	15	95.32	4.84	428	428	3	6	33.3	0.030
	16	91.21	9.35	182	191	0	2	0.0	1.000
	17	96.14	3.96	311	313	2	3	40.0	0.054
	18	93.25	7.07	1087	976	5	13	27.8	0.012
	19	-	-	69	-	-	-	-	-
	20	98.88	1.13	358	38	1	13	7.1	1.000

Proximal Promoter^5^	*LWS*	97.56	2.48	1000	1000	1	16	5.9	1.000
	*RH2Aα*	94.80	5.38	1000	1000	10	11	47.6	< 0.001*
	*RH2Aβ*	91.77	8.60	1000	1000	14	19	42.4	< 0.001*
	*RH2B*	61.35	9.40	1000	1000	15	7	68.1	< 0.001*
	*SWS1*	71.49	26.37	1000	1000	18	10	64.3	< 0.001*
	*SWS2A*	97.19	2.87	1000	1000	11	12	47.8	< 0.001*
	*SWS2B*	81.96	16.31	1000	1000	4	10	28.6	0.021

3'-UTR^6^	*LWS*	93.39	6.92	189	189	1	4	20.0	0.341
	*RH2Aα*	94.04	6.21	438	442	4	9	30.8	0.016
	*RH2Aβ*	93.26	7.06	465	460	4	11	26.7	0.027
	*RH2B*	93.15	7.18	310	319	4	4	50.0	0.002*
	*SWS1*	96.74	3.33	217	242	1	3	25.0	0.284
	*SWS2A*	98.37	1.64	123	123	0	1	0.0	1.000
	*SWS2B*	95.90	4.21	124	137	4	1	80.0	< 0.001*

^1 ^Pairwise sequence divergence between *O. niloticus *and *M. zebra*, corrected for multiple hits.

^2 ^Actual proportion of divergent TFBSs observed for *O. niloticus *and *M. zebra*.

^3 ^P-values for the Exact binomial test at a null proportion divergence = 8%. Tests marked with an asterisk (*) are significant after Bonferroni correction for multiple comparisons.

^4 ^See Additional file 6 for individual counts of each TFBS identified for the CNEs.

^5 ^See Figure 3 for individual counts of each TFBS identified for the proximal promoters.

^6 ^See Additional file 7 for individual counts of each microRNA target site identified for the 3'-UTRs.

Consistent with the high similarity of their sequences, the results of our TFBS search differed very little between *O. niloticus *and *M. zebra*. We used exact binomial tests to compare the proportion of shared and divergent TFBSs observed between *O. niloticus *and *M. zebra *to the null ratio of 92:8 (see above). Treating each TFBS independently, we counted each non-orthologous or divergent TFBS as a success, each orthologous or shared TFBS as a failure, then tested the hypothesis that the true probability of success (proportion of divergent TFBS, P_div_) was > 8%. Of 17 testable CNEs, we found that *O. niloticus *and *M. zebra *differed significantly from this null expectation at four CNEs: CNEs 3, 4, 15, and 18 (Table 3). After Bonferroni correction for multiple comparisons, however, only the results for CNE 3 remained significant (exact binomial test: divergent TFBS = 7, total TFBS = 8, P_div _= 87.5%, p < 0.001). Overall, these results did not change when we used the mean divergence of introns from each CNE's nearest down-stream opsin as a null hypothesis, except that *O. niloticus *and *M. zebra *also exhibited significant divergence at CNE 4 (divergent TFBS = 2, total TFBS = 2, P_div _= 100.0%, p = 0.001). Both CNE 3 and 4 are located upstream of the *SWS2A *opsin. For CNE 3, *O. niloticus *has 8 TFBS while *M. zebra *has only one; for CNE 4, *M. zebra *has two while *O. niloticus *has none. These results are consistent with what one might expect based on the expression of these opsins in adults, since *SWS2A *is highly expressed among *O. niloticus *adults, but is not expressed in *M. zebra *[32]. Thus, we show that *O. niloticus *and *M. zebra *have diverged significantly in the identity of their TFBS profiles for two putative *cis*-regulatory elements (CNEs 3 and 4), and differ in the presence/absence of two more (CNEs 6 and 19). Three of these CNEs (3, 4, and 6) are found upstream of the *SWS2A *opsin (Figure 1). These results offer the compelling possibility that at least some of the differences in opsin expression observed between *O. niloticus *and *M. zebra *could be due to divergence in the TFBS profiles of CNEs surrounding their opsins.

We acknowledge that our use of the overall proportion of divergent TFBS (P_div_) to detect CNEs that have undergone significant *cis*-regulatory divergence ignores many nuances of TFBS evolution, such as the overall number and kind of TFBS present in each CNE and species. But because of the small number of TFBSs found within each CNE (the average number of TFBSs found in each CNE was 5.9), it is difficult to perform robust tests of divergence in the number of binding sites for individual transcription factors. Therefore, we have summed all TFBSs into orthologous (shared) and non-orthologous (divergent) groups in order to perform phylogenetic shadowing between *O. niloticus *and *M. zebra*. However, even within these broad categories, we have only enough power that CNEs with P_div _> 25% stand out as statistical outliers, and only those with P_div _> 80% remain significant after correction for multiple comparisons. In the future we aim to perform more nuanced, sequence-based tests of *cis*-regulatory divergence in cichlids. We present these tests for *cis*-regulatory divergence as a first step in this process.

### Analysis of Proximal Promoter regions

#### Phylogenetic footprinting of opsin proximal promoters

The MultiPip plots shown in Figure 1 reveal 20 CNEs upstream of the opsins, but also show several regions of high conservation within the 5' proximal promoter of multiple opsins as well. In particular, *SWS2A, SWS2B*, and *LWS *all exhibit regions of high conservation in the first 1 kb of sequence upstream of their translation start site (TSS). For the *LWS *opsin, this region of conservation spans nearly 0.7 kb of the proximal promoter in multiple fish species, including *G. aculeatus, O. latipes*, and *T. nigroviridis *(Figure 1B). *RH2A *and *RH2A *also exhibit some small regions of high conservation just upstream of their TSSs, which probably reflect the 5'-UTR region. Additionally, the promoter upstream of *RH2B *also contains some conserved regions of repetitive sequence (Figure 1C). It is compelling that many of the opsins exhibit strong conservation of sequences within 1 kb of their TSSs, which we use to define the proximal promoter, because the true promoter regions for these genes are unknown in cichlids. However, important *cis*-regulatory sequences have been identified in close proximity to the opsin genes in other fish species. In particular, several CRX transcription factor binding sites found within 500 bp of the *SWS2 *opsin regulate the expression of this gene in *D. rerio *[41]. Therefore, the conservation we observe upstream of the *SWS2A, SWS2B*, and *LWS *opsins may indicate the presence of additional *cis*-regulatory sequences within the proximal promoters of these genes as well. A FASTA file of all opsin and non-opsin promoter sequences (see below) from *O. niloticus *and *M. zebra *is presented in Additional file 5.

#### TFBS search and phylogenetic shadowing of opsin proximal promoters

**Figure 3:**
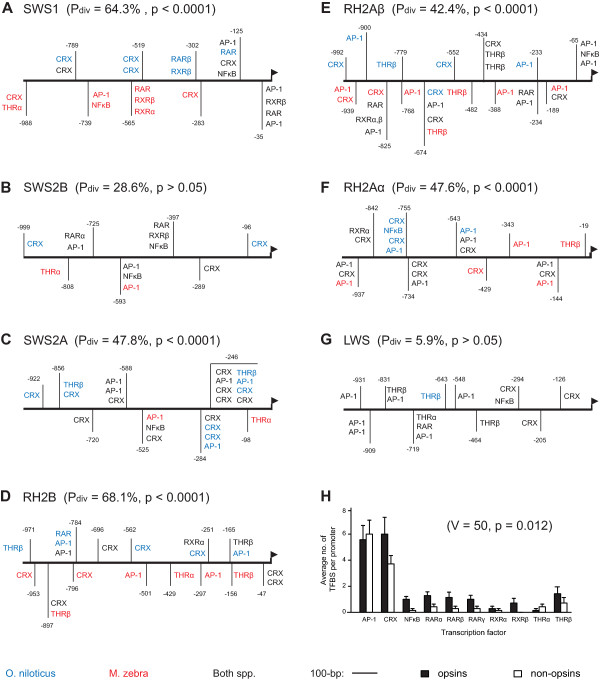
**Transcription factor binding site diversity within opsin proximal promoters**. A - G) Distribution of ten transcription factor binding sites (TFBS) in the proximal promoters of each opsin in *O. niloticus *and *M. zebra*. TFBS labelled in blue are present in *O. niloticus *only, those in red are present in *M. zebra *only, and those in black are found in both species. Sites labelled simply RAR correspond to all three retinoic acid paralogs. The orientation of factors above or below the central reference line has no special meaning, although *O. niloticus*-only sites are generally above the line, and *M. zebra*-only sites are below it. H) Comparison of the average number of binding sites for each transcription factor in the proximal promoters of the opsins and seven randomly-selected, non-opsin genes in *O. niloticus*. On average, the opsins contain significantly greater numbers of binding sites for these transcription factors compared to the non-opsin genes.

The distribution and number of TFBSs found within the proximal promoter region of each opsin was similar to those found in the CNEs. Within each opsin's proximal promoter, we found that AP-1 and CRX binding sites were nearly ubiquitous (Figure 3). Binding sites for NFκB, RARα, RARβ, RXRβ and THRβ were also common, and we once again found no binding sites for PNR and RXRγ. The absence of binding sites for PNR and RXRγ in both the CNEs and promoters may rule-out these factors as candidate *trans*-regulators of cichlid opsin expression differences; however the lack of these factors could also be due to biases in the way TESS identifies binding sites. Interestingly, we found several CRX binding sites directly upstream of the *SWS2A *and *SWS2B *opsins (Figure 3). These binding sites could potentially function as regulators of *SWS2 *opsin expression in cichlids as they do in zebrafish [41].

Pairwise sequence divergence in the proximal promoter regions was greater than for the other regions examined. The average D_xy _of the proximal promoters was 10.2% (± 3.2%), which differed significantly from the mean of CNEs (4.2%, t-test: t_23, 0.05 _= 2.48, p = 0.021), but not the introns (9.5%, t-test: t_27, 0.05 _= 0.14, p = 0.89). This result suggests that the opsin promoter regions of cichlids may exhibit greater divergence in putative *cis*-regulatory sequences than the CNEs. Indeed, we found that *O. niloticus *and *M. zebra *exhibited significant divergence in their TFBS profiles for six of the seven proximal promoters examined (Figure 3); however, following correction for multiple hypothesis testing, only five of these remained significant: *SWS1, SWS2A, RH2B, RH2A *and *RH2A *(Figure 3; see also Table 3). *O. niloticus *and *M. zebra *differ dramatically in the expression of each of these genes [32], suggesting that their divergent transcription factor profiles could explain these differences. A comparison of which TFBS differ between *O. niloticus *and *M. zebra *reveals a slight over-representation of CRX sites in *O. niloticus *(17 vs. 7), and of THRα sites in *M. zebra *(4 vs. 0) (Figure 3).

Using phylogenetic shadowing, we identified five cichlid opsins with promoter sequences that exhibit significant divergence in their binding site profiles for 12 transcription factors. We note, however, that by focusing on only these TFBSs, we potentially miss many interesting patterns of divergence in transcription factors that have not already been associated with vertebrate opsin expression. A comprehensive search of all TFBSs identified by TESS could potentially pick up these missed patterns, but such a search would be extremely cumbersome and subject to many false positives [66]. Because of their small size, TFBS motifs are likely to appear throughout the genome frequently by chance, and it is difficult to determine which are likely to be functional based on sequence matches alone. Therefore, we opted to focus on genes that are obvious candidates for analysis.

We performed an additional analysis to determine the relevance of these twelve candidate factors by comparing the number of TFBSs found for each factor within the proximal promoters of the opsins and seven randomly chosen non-opsin genes. We hypothesized that if these candidates are relevant to the control of opsin expression in cichlids, then we should find a significantly greater number of TFBSs for each factor upstream of the opsin genes compared to the non-opsin genes. Indeed, we found that the opsins contain a greater number of binding sites for eight out of ten factors compared to the randomly-chosen non-opsin genes (Wilcoxon paired signed-rank test: V = 50, p = 0.0124; Figure 3H). The non-opsin promoters contained higher mean numbers of TFBSs for AP-1 and THRα only. This result suggests that the proximal promoters of the opsins are significantly enriched for the binding sites of transcription factors that influence vertebrate opsin expression. This enrichment also suggests that polymorphisms in these regions could conceivably lead to functional differences in transcription factor binding and opsin expression. However, we note that we found no significant correlation between distance matrices of the opsins based on these TFBS profiles and their expression in developing *O. niloticus *(data not shown). This additional result suggests that, although binding sites for many of these candidate transcription factors may be over-represented in the promoters of opsins, they do not predict which opsins are co-expressed in African cichlids.

The search parameters we have chosen aim to identify TFBSs with high confidence while still accounting for the observation that many transcription factors exhibit degenerate binding of DNA motifs [67,68], and can bind these motifs in an orientation-independent manner [69,70]. We are currently performing a quantitative genetic analysis of many markers located across the genome in order to identify other loci and transcription factors that may be associated with cichlid opsin expression. This quantitative genetic analysis should provide an unbiased search for additional transcription factors that may influence cichlid opsin expression.

### Analysis of opsin 3'-UTRs

#### Phylogenetic footprinting of opsin 3'-UTRs

In addition to mutations within conserved non-coding elements and 5' promoter regions, polymorphisms within 3'-UTRs can also act as *cis*-regulatory alleles [16,17]. These polymorphisms affect gene expression by altering the binding of miRNAs in a manner analogous to how mutations within TFBS can alter gene expression, except that miRNAs inhibit gene expression post-transcriptionally. Our phylogenetic footprinting analysis reveals that every opsin exhibits some conservation of the 50 - 100 bp region found directly downstream of the opsin coding sequences (Figure 1). Generally, this conservation is strongest between *O. niloticus, O. latipes*, and *G. aculeatus*, reflecting the close phylogenetic relationship among these species. For *RH2A*, the 3' conserved region extends nearly 700 bp past the end of the coding region. Initially, these results suggest that the opsin 3'-UTRs of cichlids will be highly conserved, reflecting the strong evolutionary constraint on UTR sequence and function seen in both flies and humans [16,71]. However, a recent survey of polymorphisms affecting miRNA target sites in cichlids found that the 3'-UTR of some genes may in fact be under divergent selection in African cichlids [72]. Therefore, we searched the 3'-UTRs of the opsins for target sites corresponding to known fish miRNAs.

Of the 30 known miRNA targets we searched for in cichlids (see below), we found at least one target site in each opsin 3'-UTR that was conserved among cichlids and other teleosts (Table 4). Many of these conserved sites are expressed within the retina of vertebrates and play a role in retinal development [73-76]. For example, dre-miR-217, dre-miR-181a, and dre-miR-23b are all integral to the development and maintenance of the zebrafish retina [77-79], while dre-miR-96 and dre-miR-182a are sensory organ-specific [76]. Only one conserved site that was found in cichlids and other teleosts differed between *O. niloticus *and *M. zebra*. A target for dre-miR-722, found downstream of the *LWS *opsin in *O. niloticus *and the pufferfish (*Takifugu rubripes*), is missing in the orthologous 3'-UTR from *M. zebra *due to a single nucleotide polymorphism (SNP). However, the two conserved target sites for dre-miR-722 and dre-miR-728 are both found within the 3'-UTRs of several Lake Victorian cichlids (data not shown). Like *O. niloticus*, Lake Victoria's cichlids express the long wavelength opsin palette as adults [80], possibly indicating that these factors play a role in *LWS *expression. If we interpret evolutionary conservation as an indication of function, we believe the conserved sites listed in Table 4 represent those miRNA target sites that are most likely to regulate opsin expression in African cichlids. The sequences of all *O. niloticus *and *M. zebra *opsin 3'-UTRs are available in Additional file 5.

**Table 4:** Conserved microRNA target sites within the 3'-UTRs of each opsin in *O. niloticus *and *M. zebra*

Opsin	miRNA	Target	Conserved^1^	Function and expression	Ref(s)
*SWS1*	miR-725	TGACTGAG	GA	Expressed in fins	[55]
*SWS2B*	miR-217	ATGCAGTA	GA	Alters *PTEN *exp.; found in eye	[75,78]
*SWS2A*	miR-181a	AGAATGTA	DR	T-cell regulation; found in eye	[75,79]
*RH2B*	miR-23b	TATGTGAA	TR	Ganglion apoptosis; found in eye	[77,116]
*RH2Aα*/*β*	miR-96	TTGCCAAA	OL	Sensory organ specific; found in eye	[76,117]
	miR-182a	TTGCCAAA	OL	Sensory organ specific; found in eye	[76,117]
*LWS*	miR-728	TTTAGTAA	GA,TN,TR	Unknown; found in eye	[55]
	miR-722*	GCAAAAAA	TR	Unknown; found in eye	[55]

^1 ^Other fish species in which this target site is also found: GA = stickleback (*G. aculeatus*), DR = zebrafish (*D. rerio*), TR = fugu (*T. rubripes*), TN = pufferfish (*T. nigroviridis*); OL = medaka (*O. latipes*)

* This site present in *O. niloticus *only

#### microRNA target search and phylogenetic shadowing of opsin 3'-UTRs

We searched the 3'-UTRs of each opsin in *O. niloticus *and *M. zebra *for target sites corresponding to known fish miRNAs [54]. In all, we identified 84 predicted target sites matching 30 known miRNAs from cichlids and *D. rerio *(Additional file 7). Like the CNEs and promoter regions analyzed earlier, all 3'-UTR sequences generally exhibited high similarity between *O. niloticus *and *M. zebra*. The average pairwise divergence (D_xy_) for *O. niloticus *and *M. zebra *3'-UTRs was 5.2% (± 1% s.e.). This small level of divergence is very similar to the level observed for opsin coding sequences, though it did not differ from the average D_xy _of introns (9.5%, t-test: t_27, 0.05 _= 1.33, p = 0.196). Consequently, the results of our miRNA target search were once again very similar for *O. niloticus *and *M. zebra*, especially for those sites conserved in other fishes as well (Additional file 7; Table 4, see above). However, we still found that *O. niloticus *and *M. zebra *differed significantly in the proportion of divergent and shared miRNA target sites for the 3'-UTRs of the *RH2B *and *SWS2B *opsins (exact binomial tests: *RH2B*, divergent miRNA sites = 4, total miRNA sites = 8, P_div _= 50.0%, p = 0.002; *SWS2B*, divergent miRNA sites = 4, total miRNA sites = 5, P_div _= 80.0%, p < 0.001; see Table 3). These results did not change when we altered the null hypothesis to reflect the divergence of each opsin's intronic sequence (data not shown). For *RH2B*, we found that *M. zebra *exhibited four unique target sites for miRs-101, 144, 196, and 2184. For *SWS2B, M. zebra *had unique targets for miRNAs-194 and 23, while *O. niloticus *had targets for miRNAs -92 and 137. *RH2B *is strongly differentially expressed in these two species, while *SWS2B *is only expressed in some adults of *O. niloticus *[81]. Thus, we not only identified at least 8 conserved--and perhaps core--miRNA target sites in the 3'-UTR of each cichlid opsin (Table 4), we also found that *O. niloticus *and *M. zebra *are significantly divergent in at least two of these regions (*SWS2B *and *RH2B*).

It is important to note that most miRNA target sites we identified in the 3'-UTRs of the cichlid opsins correspond to miRNAs that are expressed in the vertebrate retina (Additional file 7). Of sites corresponding to 30 different miRNAs, 22 (73%) correspond to miRNAs expressed within the retinas of fish, mammals, or amphibians (Additional file 7). Notably, however, we did not find any miRNA target sites that correspond to miR-726, the miRNA found upstream of the *LWS *opsin and encoded by CNE 7a (see Figure 2). Further, many of the conserved and non-conserved miRNA target sites we identify also correspond to miRNAs associated with retinal development (for example, dre-miRs 23, 92, 722, and 194) [76,82,83], and miR-129 is also associated with retinoblastoma in humans [84]. Given that *O. niloticus *and *M. zebra *differ dramatically in their developmental patterns of opsin gene expression, it is interesting to speculate that these miRNAs could contribute to the developmental differences in opsin expression observed between these and other African cichlid species [32,85]

In the present study we have focused on miRNA target sites found within the 3'-UTR of the cichlid opsins, but miRNA cleavage of messenger RNAs by binding to sites within core messenger RNA sequences has also been demonstrated in humans and plants [86,87]. It is still not clear whether miRNAs regulate gene expression more often by binding to the 3'-UTR or messenger RNA sequence, although a review by Bartel [88] suggested that translational repression by binding to UTR sequences is more prominent. Finally, we note also that the cellular machinery cannot distinguish between functional and non-functional miRNA target sites based on their evolutionary conservation in other species, as we do here [88] (see Table 4). However, given that scans for miRNA target sites can have a high rate of false positives, evolutionary conservation is currently the best way to avoid high error rates and to infer function. The fact that we identified a high percentage of target sites that correspond to miRNAs found within the vertebrate eye suggests that many of these sites are not false-positives; therefore, it is plausible that they may actually function to regulate opsin expression in cichlids. In the future we will determine whether these and other miRNAs are actually expressed in the retinas of African cichlids. If so, then heterologous reporter assays could be used to verify what role divergence in miRNA target sites may play in the evolution of cichlid opsin expression [89,90].

### Phylogenetic shadowing among the cichlids of Lake Malawi

#### Resequencing and analysis of LWS-LCR, opsin promoters, and 3'-UTRs

Two broad goals of this study have been to (1) identify potential *cis*-regulatory sequences surrounding the opsin gene arrays of African cichlids (phylogenetic footprinting), and (2) identify those sequences whose divergence may explain patterns of differential opsin gene expression among African cichlids (phylogenetic shadowing). For both goals we have relied on sequenced BAC clones of *Oreochromis niloticus *and *Metriaclima zebra*--two species that have BAC libraries available, but that also differ dramatically in their evolutionary age (~18 MY [33]) and adult and developmental patterns of opsin expression [32]. Therefore, as a final goal, we wanted to determine whether the candidate *cis*-regulatory sites we identified via phylogenetic shadowing also vary among a more closely related (~2 MY [33]) panel of 18 cichlid species from Lake Malawi. Although much more closely related to *M. zebra *than *O. niloticus*, adults of these species exhibit the same opsin expression patterns as adult and juvenile *O. niloticus *[23,24,26]. Our panel included one individual from six species for each of the three adult opsin expression palettes observed among Lake Malawi's cichlids (short-, middle-, and long-wavelength sensitive) (see Additional file 8 for a list of the species used). The regions we re-sequenced included the proximal promoters upstream of the *SWS1, SWS2A, SWS2B, RH2B*, and *LWS *opsins (highlighted in blue in Figure 1), the LWS-LCR (CNE 7), and the 3'-UTRs of the *SWS2B *and *LWS *opsins. After sequencing, we examined these regions for levels of interspecific polymorphism and performed a test of association for *cis*-regulatory alleles.

**Figure 4:**
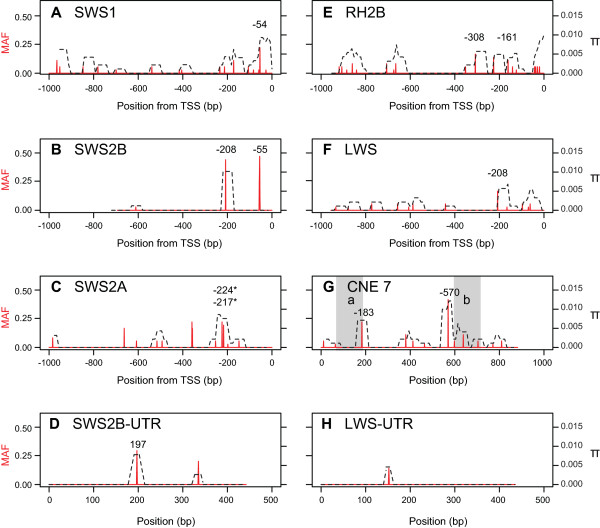
**Interspecific polymorphism in eight putative *cis*-regulatory regions from 18 Lake Malawi cichlid species**. A - H) Minor allele frequency (MAF; in red) and nucleotide diversity (π; in black) calculated in a sliding window across the proximal promoter regions of five opsins (A - E), CNE 7 (LWS-LCR) (F), and two opsin 3'-UTRs (G - H) using 18 Lake Malawi cichlid species. Numbers above peaks of MAF and π denote the position of SNPs analyzed for allelic-association with opsin expression (see Table 6); asterisks (*) denote polymorphisms that interrupt CRX binding sites.

Although the 18 Lake Malawi cichlid species we use have been previously characterized with regard to opsin gene expression, we confirmed these gene expression results by measuring the expression of each opsin in all species via RT-qPCR (see Additional File 8 for opsin expression results). These expression results were highly concordant with previous measurements [26]. Following qPCR, we re-sequenced the entire 1 kb region upstream of both the *SWS1 *and *SWS2A *opsins, 956 bp upstream of the *LWS *opsin, 951 bp upstream of the *RH2B *opsin, and 694 bp upstream of the *SWS2B *opsin. We also re-sequenced 900 bp surrounding the LWS-LCR (CNE 7) and 450 bp downstream of the *SWS2B *and *LWS *opsins. As expected given the young age of Lake Malawi cichlids, we found that all regions were highly conserved among the species sampled. Overall, we identified fewer than 15 single nucleotide polymorphisms (SNPs) and insertion/deletions (indels) per region examined (Table 5). In each case, most SNPs were found in only one individual. Other diversity statistics--including the total number of segregating sites (S), total number of singletons (s), number of haplotypes (H), nucleotide diversity (π), sequence conservation (C), and Tajima's D (T_D_)--also indicate low levels of polymorphism, despite our use of alternate species and genera as sampling units (see Additional file 8 for a list of all polymorphisms found among the 18 species sampled). Nevertheless, following a sliding window analysis of nucleotide diversity (π) and minor allele frequency (MAF), we were able to identify several peaks of relatively highπ and MAF within each region (Figure 4). These peaks correspond to SNPs and indels segregating at high frequency within the species and genera sampled, and therefore represent potential *cis*-regulatory alleles.

**Table 5:** Polymorphism statistics for 8 candidate *cis*-regulatory regions in 18 Lake Malawi cichlid species

Opsin	Length (bp)	*S*^1^	s^2^	H^3^	π^4^	C^5^	*T_D_^6^*	CRX^7^
*SWS1*	1000	16	5	17	0.0020	0.983	-1.4424	1
*SWS2B*	694	2	1	3	0.0008	0.997	0.2951	0
*SWS2A*	1000	7	1	6	0.0010	0.992	-1.1518	2
*RH2B*	950	17	3	15	0.0022	0.982	-1.1050	2
*LWS*	956	12	2	11	0.0012	0.987	-1.2394	0
CNE 10	882	12	1	10	0.0021	0.986	-0.2311	0
*SWS2B *UTR	442	2	0	4	0.0013	0.995	0.4486	NA
*LWS *UTR*	436	1	0	2	0.0006	0.998	0.0298	NA

^1 ^Total number of segregating sites

^2 ^Total number of segregating sites that are singletons

^3 ^Total number of haplotypes

^4 ^Nucleotide diversity

^5 ^Sequence conservation

^6 ^Tajima's D

^7 ^Total number of segregating sites that interrupt predicted CRX binding sites

* Statistics presented for in/del polymorphism

Several peaks of relatively high nucleotide diversity and MAF correspond to polymorphisms within predicted CRX binding sites, but none correspond to any other TFBS or miRNA target sites (Table 5). Specifically, two peaks of π and MAF located -217 and -224 bp upstream of the *SWS2A *translation start site (TSS) correspond to a single SNP and 8 bp indel that both disrupt putative CRX binding sites. The 8 bp indel located at *SWS2A*-217 completely eliminates the CRX binding site in several species (Additional file 8). We identified at least three other polymorphisms upstream of the *SWS1 *and *RH2B *opsins that also disrupt CRX binding sites--each present in only a single species--but no polymorphisms that interrupt the binding sites of any other candidate transcription factors. One peak of nucleotide diversity at position 183 of CNE 7 (see Additional file 5 for this sequence) corresponds to a SNP within the miRNA miR-726; however, this mutation does not occur within the mature miRNA sequence. Finally, we found only three polymorphisms total within the 3'-UTR of both the *SWS2B *and *LWS *opsins (Figure 4; Additional file 8), none of which interrupted predicted miRNA target sites. The polymorphism that segregates between *O. niloticus *and *M. zebra *within the *LWS *3'-UTR was fixed in all Lake Malawi cichlid species (see Table 4). Thus, few of the polymorphisms we identify in the putative *cis*-regulatory sequences of the opsins are predicted to alter opsin expression among 18 Lake Malawi cichlid species. However, since mutations within transcription factor binding sites have been shown to alter gene expression [91], our results suggest that polymorphisms within CRX TFBSs could contribute to the differential patterns of *SWS2A *expression observed among Lake Malawi cichlids.

#### Association between polymorphisms and cichlid opsin expression

To test this hypothesis, we performed allelic association tests between these and other SNPs underlying peaks of nucleotide diversity and high MAF (see Figure 4) with the expression of their nearest downstream opsin (Table 6). Three polymorphisms (*SWS2A*-217, *RH2B*-161, CNE 7-570) exhibited significant or marginally non-significant associations with the expression of their downstream opsins (Table 6); however only *RH2B*-161 is significant following Bonferroni correction for multiple comparisons (t-tests: *RH2B*-161: t_17, 0.05 _= 3.447, p = 0.0036). Despite this limitation, we believe these preliminary results are compelling since all three polymorphisms occur on the same linkage group (LG 5) believed to contain a *cis*-regulatory element that modulates cichlid opsin expression, and all three are associated with opsins whose expression is significantly associated with this QTL in African cichlids [29]. *SWS2A*-217 obliterates a CRX binding site in numerous cichlids, and polymorphisms affecting CRX binding sites have been shown to modulate *SWS2 *opsin expression in zebrafish [41]. CNE 7-570 is found very near the LWS-LCR and could potentially affect LCR binding. It is therefore possible that all three alleles acts as, or are linked to, *cis*-regulatory elements that modulate opsin expression in cichlids.

**Table 6:** Results of allelic association between SNPs underlying peaks of nucleotide diversity and opsin expression in 18 Lake Malawi cichlid species

Polymorphism distance from TSS	Type	MAF^1^	*r*^2^	t-value	P-value
SWS1 -54	C*T	0.222	-0.279	-0.911	> 0.05
SWS2B -208	C*T	0.417	< 0.001	0.003	> 0.05
SWS2B -55	1 bp indel	0.444	0.240	0.789	> 0.05
SWS2A -224*	C*T	0.222	0.127	1.037	> 0.05
SWS2A -217*	8 bp indel	0.194	0.392	1.841	0.087
RH2B -308	C*G	0.167	-0.245	-0.893	> 0.05
RH2B -161	C*T	0.111	0.263	3.447	0.004
LWS -208	C*T	0.167	0.355	1.002	> 0.05
CNE-7 183	A*T	0.222	0.055	-0.673	> 0.05
CNE-7 570	C*T	0.417	0.608	2.237	0.041
SWS2B-UTR 197	A*C	0.306	0.349	1.264	> 0.05

* These polymorphisms interrupt CRX transcription factor binding sites

We acknowledge that the sample sizes we use for phylogenetic shadowing among Lake Malawi's cichlids are small and at best provide a weak test for *cis*-regulatory alleles associated with opsin expression. Additionally, we use cross species and genera comparisons for an analysis that is generally based on individual variation within populations. However, Lake Malawi cichlids are extremely similar at the genetic level and share many ancestral polymorphisms [92]. For this reason, genetic analyses across cichlid species are analogous to within-species polymorphism studies in other vertebrates, such as chimps and humans [72,92]. Additionally, recent work in cichlids has successfully used cross-species comparisons to fine-map *cis*-regulatory alleles underlying pigmentation differences, so long as these differences have a common origin among the different species sampled [93]. It is hard to predict which traits will have a common origin among different African cichlid species, as previous work [94] suggested that the pigmentation trait mapped in Roberts et al. [93] had evolved several times. Our recent work reconstructing the evolution of opsin regulatory changes in cichlids revealed that the three opsin expression palettes have evolved repeatedly among cichlids in Lakes Tanganyika and Malawi [27], but it is still unclear whether or not the three palettes have a common origin among Lake Malawi's cichlids. But despite our small sample size, we have found some evidence of binding site turnover in CRX binding sites within the 5' promoters of Lake Malawi cichlids, but no evidence of turnover in other candidates TFBS or miRNA target sites. Additionally, we also identified three putative *cis*-regulatory polymorphisms associated with *SWS2A, RH2B*, and *LWS *opsin expression. Although very preliminary, these results offer compelling candidates for additional functional and association analyses between more closely related cichlid populations and species.

### The search for *cis*-regulatory sequences

**Figure 5:**
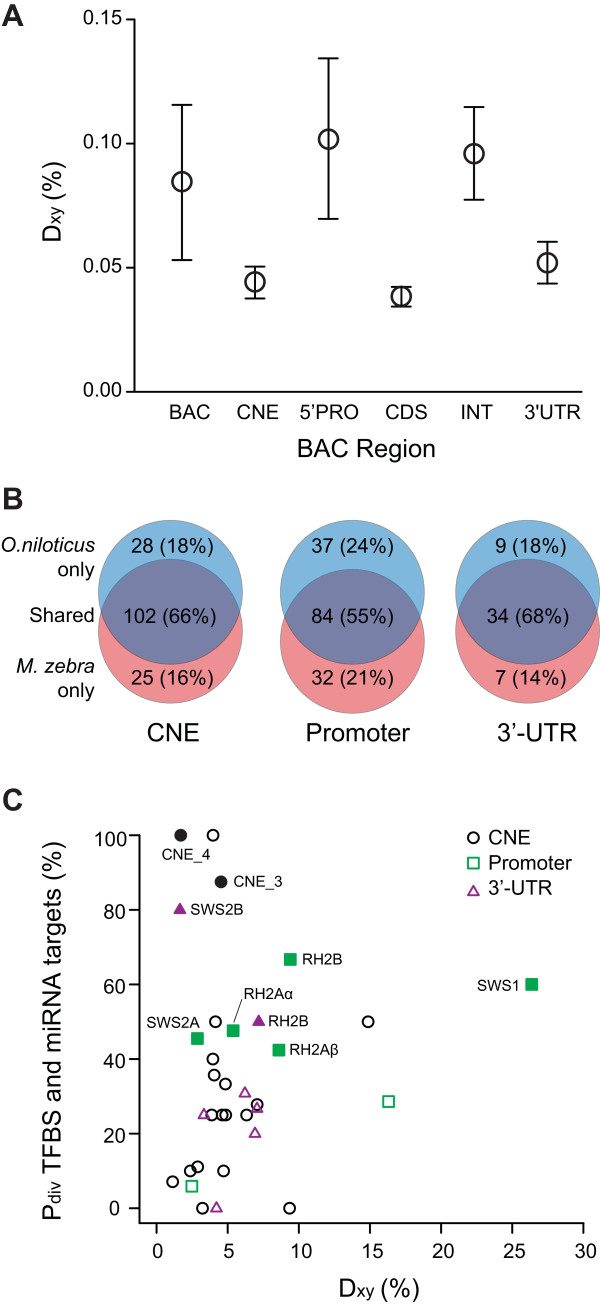
**Divergence among coding and non-coding regions in *O. niloticus *and *M. zebra *BAC sequences**. A) Pairwise sequence divergence (D_xy_) between *O.niloticus *and *M. zebra *for different coding and non-coding regions of the opsin-containing BACs. Average Jukes-Cantor-corrected D_xy _is higher among 5' proximal promoter regions for each opsin. B) Venn diagram of proportion of shared and divergent TFBS and microRNA target sites among non-coding regions examined in this study. Opsin promoter regions exhibit slightly elevated proportions of divergent sites compared to either CNEs or 3'-UTRs. C) Comparison of proportion divergent TFBS/miRNA target sites (P_div_) and pairwise sequence divergence (D_xy_). Non-coding sequences with elevated P_div _do not necessarily exhibit increased D_xy_, even among proximal promoters regions. Filled points are those sequences with P_div _values that differ significantly from 8% (see also Table 3).

*Cis*-regulatory sequences may reside many kilobases away from the genes they regulate, as in the case of enhancer or repressor elements; or they may be found very near their genetic targets, as in the case of promoter elements and UTRs. Given this diversity, is it possible to predict which non-coding regions are most likely to contain functional *cis-*regulatory alleles? If we accept estimates of pairwise sequence divergence (D_xy_) as indicative of those regions most likely to contain functional opsin regulatory alleles, then our estimates of D_xy _between *O. niloticus *and *M. zebra *suggest that the proximal promoter regions are most likely to contain *cis*-regulatory alleles that alter opsin expression (Figure 5A; see also Additional file 9 for a list of D_xy _values for every region examined). The opsin promoters exhibit the highest levels of pairwise sequence divergence of all coding and non-coding regions examined, and also contain more sequences with divergent TFBS profiles (Figure 3; Table 3), and putative regulatory alleles (Table 6). However, this conclusion is undoubtedly influenced by what could be a naive choice of promoter sequences (the true functional opsin promoter regions have not yet been identified in cichlids and may be more highly conserved), increased length of the promoter sequence relative to other regions analyzed (we analyzed 1 kb for each promoter versus ~ 400 bp for each CNE and UTR), and the increased power to detect significant divergence from null expectations afforded by the large number of TFBS found within the proximal promoters (we found ~ 22 TFBS within each promoter versus ~ 6 TFBS/miRNA target sites within each CNE and UTR).

If the overall proportion of divergent TFBS/miRNA target sites (P_div_) is used to identify those non-coding regions most likely to contain functional *cis*-regulatory alleles, the proximal promoter regions still exhibit the highest proportion of divergent regulatory regions, although the advantage is only slight. Only about 55% of TFBS are shared between *O. niloticus *and *M. zebra *promoters, while 45% are divergent (Figure 5B). In contrast, the CNEs and 3'-UTRs exhibit lower (and very similar) proportions of shared versus divergent TFBS/miRNA target sites (~ 67% shared and ~ 33% divergent; Figure 5B). In this case, it is difficult to confidently conclude that 5' promoter regions are more likely to contain functional alleles that regulate opsin expression, although the data are suggestive. When both pairwise divergence and the proportion divergent TFBS/miRNA target sites are taken into account, we find that regions that exhibit statistically significant divergence are not necessarily those regions that exhibit greater pairwise sequence divergence (Figure 5C). In fact, the regions with the highest P_div _also exhibit some of the lowest D_xy _values. This result suggests that the increased number of statistically divergent promoter regions we observe is not a function of sequence divergence, but rather increased statistical power afforded by the greater length of the sequences surveyed and the greater number of TFBS found.

Additionally, our results show that the majority of the non-coding regions examined exhibit P_div _values near 37%, with a median of 30% (Figure 5C). This observation suggests that the 8% divergence criterion we used as null model for evolutionary divergence is likely too low and also suggests that our power for many regions was inadequate due to the small number of TFBS or miRNA target sites identified (see above). But even when a more liberal null divergence value of 30% is used, our results largely remain consistent: *O. niloticus *and *M. zebra *still exhibit significant divergence in their TFBS and miRNA target profiles for CNEs 3 and 4 (located near the *SWS2A *opsin), the proximal promoters for *RH2B *and *SWS1*, and the 3'-UTR for *SWS2B *(p < 0.05; see Table 3 for P_div _values).

Finally, we note that many putative regulatory regions identified in our opsin-containing BACS are highly conserved among many phenotypically diverse cichlid species from Lake Malawi, as well as between the ~18 MY divergent *Oreochromis niloticus *and *Metriaclima zebra*. This conservation suggests that *trans*-acting factors may also play an important role in generating evolutionary changes in cichlid opsin expression. For example, in both yeast and humans, interspecific differences in gene expression are primarily the result of *trans*-regulatory factors [95,96]. And although *cis-*regulatory alleles contribute more to interspecific differences in gene expression among several *Drosophila *species, *trans-*acting alleles generally contribute to these differences as well [97]. Coding mutations within *trans-*acting transcription factors can act in a modular fashion, thereby mitigating negative pleiotropic effects [98], and these mutations may still affect gene expression even when the sites they bind remain conserved [99], as many of the TFBSs we examine are. Also, in addition to the putative *cis*-regulatory factors associated with *SWS2B, SWS2A*, and *RH2B *opsin expression in cichlids, Carleton et al. [29] also identified one *trans*-acting locus in the same cross, as well as another *trans*-acting locus in a separate cross. These two loci, located on cichlid LGs 13 and 4, do not occur in linkage with the cichlid opsins and explain a higher portion of the variance in opsin expression than the single *cis*-associated factor on LG 5 [29]. Whether these sites represent transcription factors, miRNAs, or other *trans*-acting binding sites is unknown, but several good candidate genes are located in these regions. Future work will aim to map and characterize these putative *trans*-regulatory regions in a variety of cichlid taxa.

## Conclusions

Mutations within *cis*-regulatory regions are compelling candidates for the adaptive evolution of gene expression [3]. Here we generated and surveyed non-coding sequences surrounding the opsin gene arrays of two African cichlids, *Oreochromis niloticus *and *Metriaclima zebra*. This study is the first to systematically survey the cichlid opsins for putative *cis-*regulatory sequences, and our results suggest that these regions could potentially contribute to variation in cichlid opsin expression. The results of our study reveal:

(1) The presence of numerous conserved non-coding elements located up- and downstream of the opsins that may function as regulators of cichlid opsin expression, including a retinal miRNA and one known opsin enhancer (LWS-LCR). African cichlids were divergent in two of these (CNEs 3 and 4, both located upstream of the *SWS2A *opsin).

(2) Significant divergence and enrichment of transcription factor binding sites within the proximal promoter of five of the seven opsins (*SWS1, SWS2A, RH2B, RH2A*, and *RH2A*).

(3) Numerous target sites for retinal and sensory organ-specific miRNAs within the 3'-UTR of each opsin. African cichlids were divergent in two of their opsin 3'-UTRs (*SWS2B *and *RH2B*).

(4) The presence of several candidate *cis*-regulatory alleles located within the promoters of the *RH2B *and *SWS2A *opsins, as well as one near the LWS-LCR (CNE 7).

Future work will aim to further characterize these candidate *cis*-regulatory sequences, as well as to identify candidate *trans*-acting alleles. Given that spectral sensitivity and opsin expression in vertebrates can be influenced by coding mutations [26,80,100], *trans*-regulatory mutations [29], *cis*-regulatory mutations [41], and possibly miRNAs as well, cichlids may represent an ideal system in which to examine how these various molecular mechanisms interact to influence the evolution of visual system diversity in vertebrates.

## Methods

### Sequencing and assembly of BAC clones

We isolated clones containing the opsin genes from BAC libraries of two African cichlids, *Oreochromis niloticus *[30] and *Metriaclima zebra *[31]. For *O. niloticus*, we used PCR to screen pooled clones from the T3 and T4 libraries [30]. Primers used for these screens were: *SWS1 *(*F*: TACCTGCAGGCTGCCTTTAT; *R*: CTCGCATGGAGGCTAAGAAC), *RH2A *(*F*: GCAGACCCGATCTTCTTCAA; *R*: AGCAGACGTGATTGTGATGG), and *LWS *(*F*: TCCTGTGCTACCTTGCTGTG; *R*: ACAACGACCATCCTGGAGAC). We first chose 10 super-pools, each covering 10% of the entire 35,000 pooled clones, and screened them for opsin-positive plates. We then screened row and column pools from the plates with positive results to identify the exact clones containing the opsins. Fingerprinted contigs (FPCs) corresponding to the positive clones were identified and all clones in the contig were PCR tested for the opsins (see Additional file 10). Contig geometries were confirmed by end sequencing the BACs, designing primers, and PCR testing (Additional file 10). Based on the resulting alignments, one clone for each opsin array was selected for sequencing.

DNA from the selected clones was prepared using the Qiagen^® ^MaxiPrep Plasmid Purification kit following the manufacturer's protocols. The *O. niloticus *clones were sent to the Joint Genome Institute (JGI) for ABI-Sanger sequencing. Shotgun libraries were prepared and 4 × 384-well plates were sequenced using ABI technology in both forward and reverse directions. The resulting reads were base-called and assembled with phred [101] and phrap [102]. Additional reads for the *SWS1*-containing clone were generated using 454 Life Sciences technology [103]. We performed two different sequencing runs for this clone, assembled them into contigs, and combined them with the JGI ABI reads in Sequencher v4.9 (Gene Codes Corporation, Inc.). This resulted in several large but non-overlapping contigs. To finish joining these contigs we used BLAST [104] and Pipmaker [45] to identify and align the largest contigs to orthologous genomic regions from the genomes of other teleost fish (for an example see Additional File 2). Based on these alignments we designed PCR primers to sequence across the gaps to join the contigs.

For *M. zebra *we screened high-density BAC array filters using filter hybridization [31]. This search utilized PCR probes generated from *M. zebra *retinal cDNAs that were labeled using the ECL Nucleic Acid Labelling and Detection Kit (Amersham Biosciences). We obtained three clones from these arrays and confirmed that they contained the opsins via PCR as detailed above. DNA for these clones was prepared using the Qiagen^® ^MaxiPrep kit following the manufacture's protocols. BAC clones were sized by pulsed field gel electrophoresis following digestion with NotI. We then sent the purified, sized samples to 454 Life Sciences (Branford, CT) for sequencing on the GS20. We performed two sequencing runs on the *SWS1 *and *LWS*-containing clones, but only one for the clone containing *RH2A*. All runs utilized a quarter plate. Due to the length of the 454 reads, the resulting sequences formed more, but smaller contigs relative to *O. niloticus*. To finish joining these contigs we aligned the largest (> 5 kb) contigs to the finished *O. niloticus *BAC sequences in Sequencher v4.9 and once again designed PCR primers to sequence across the gaps. We annotated the BAC sequences for both *O. niloticus *and *M. zebra *using BLAST [104].

Finally, we performed a global alignment of each BAC from *O. niloticus *and *M. zebra *in the program wgVISTA [105]. We measured sequence similarity and divergence across each BAC using the phylip program dnadist, implemented in the Mobyle online bioinformatics server [106]. When measuring pairwise sequence divergence (D_xy_), we used the Jukes-Cantor nucleotide model to correct for multiple hits. We repeated these measurements for each of the CNEs, promoter regions, and 3'-UTRs. We compared D_xy _among each of these regions and the entire BAC sequences using t-tests implemented in the statistical software package R v2.10.0 [107]. Prior to performing all tests, we transformed the D_xy _scores by log_10 _in order to meet the assumption of normality of errors.

### Phylogenetic analyses

We generated phylogenies of the teleost *RH2 *and *SWS2 *opsins in order to identify orthologous opsins among the focal fish genomes examined. We accessed all relevant opsin sequences from the genome assemblies listed above via BLAT. We aligned both opsin data sets using the E-INS-i strategy of the multiple alignment program MAFFT v6.0 [108] and then chose an appropriate model of nucleotide substitution via the program jModelTest v0.1.1 [109]. This model was TIM3ef+G for both the *RH2 *and *SWS2 *alignments. We then used this model and the corresponding parameters estimated by jModelTest to generate Neighbor-Joining trees for the opsins with Maximum Likelihood-corrected distances. For the *RH2*/*SWS2 *datasets, these parameters included the nucleotide substitution rate matrix (A-C: 0.601/0.617; A-G: 1.470/1.734; A-T: 1.00/1.00; C-G: 0.601/0.617; C-T: 2.729/2.877; G-T: 0.599/0.155) and the shape of the gamma distribution (0.507/0.577). We measured the nodal support of these trees with 1000 bootstrap replicates. We rooted both trees using the *LWS-1 *opsin of zebrafish.

### Identification of conserved non-coding elements

We used phylogenetic footprinting [18] to identify putative *cis*-regulatory elements by searching for conserved non-coding elements (CNEs) surrounding the opsin gene arrays. To do this, we identified 100-300 kb regions of orthology between the *O. niloticus *BAC sequences and the genome assemblies of four teleost fishes using BLAT and the UCSC genome browser. The additional genomes were stickleback (*Gasterosteus aculeatus*, Broad Institute v1.0, February 2006), medaka (*Oryzias latipes*, National Institute of Genetics and the University of Tokyo v1.0, October 2005), pufferfish (*Tetraodon nigroviridis*, Geoscope and Broad Institute v7, February 2004), and zebrafish (*Danio rerio*, Trust Sanger Institute zv8, December 2008). We then determined the location of known opsin genes and examined synteny across these regions via DOT plots generated in the program PipMaker [45] (for an example see Additional File 2). Regions of high synteny surrounding the opsins were then identified using MultiPipMaker [46]. We defined a CNE as any region ≥ 50 bp long that was conserved (> 60% sequence identity) between *Oreochromis niloticus *and at least one other teleost species (*Oryzias latipes, Gasterosteus aculeatus*, and *Tetraodon nigroviridis*). In each case, we attempted to analyze as many CNEs as possible, but acknowledge that some small regions may have been missed.

### Profiling of transcription factor binding sites and Phylogenetic shadowing

We identified binding sites within each CNE as well as the proximal promoters located approximately 1 kb upstream of each opsin's translation start site using the Transcription Element Search System, TESS v6.0 [63]. We altered the default search parameters of TESS by changing the minimum log-likelihood ratio score from 12 to 9. We then limited our search results to high quality matches by accepting only those hits that met three criteria: (1) a log-likelihood (L_a_) score ≥ 9.0, (2) a ratio of the actual log-likelihood score to the maximum possible log-likelihood (L_q_) score ≥ 80%, and (3) a probability value for the log-likelihood score (L_pv_) < 0.05. Although TESS can potentially identify binding sites for many different transcription factors, we were primarily interested in those factors that have been shown to influence opsin expression in fish and other vertebrates (Table 1). Following the automated search in TESS, we manually searched the lists for duplicate sites at each position, and removed them prior to further analysis.

For phylogenetic shadowing, we analyzed the number of shared and divergent transcription factor binding sites found in each CNE and opsin proximal promoter from *O. niloticus *and *M. zebra*. We counted the total number of binding sites orthologous in both species, as well as those that were found in only one species or the other. We calculated the proportion of divergent TFBSs (P_div_) as (D/(D+S))*100, where D is the number of divergent TFBS and S is the number of shared sites. We compared the observed proportion of divergent sites to the null proportion suggested by the global sequence similarity of the *O. niloticus *and *M. zebra *BACs (92% versus 8%). We tested the independence between these observed and expected proportions using exact binomial tests [110] implemented in the R statistical software package. To control the Type I error rate for each region examined, we calculated Bonferroni-corrected p-values for all tests in R. For phylogenetic shadowing between *O. niloticus *and *M. zebra*, the corrected significance threshold was α = 0.05/31 = 0.0016.

Finally, we also compared the average number of binding sites for each transcription factor between the proximal promoters of the *O. niloticus *opsins and seven randomly chosen, non-opsin genes from a draft assembly of the *O. niloticus *genome (available at http://www.BouillaBase.org; accessed October 2010). These genes were *ACTG1, AMPD3, DHCR7*, ENSGAC000000020282, *IGFALS, KCNJ9*, and *REEP1*. Proximal promoters from these randomly chosen sequences were identified based on comparison of the *O. niloticus *genes with orthologous regions from the stickleback genome. Comparison of the average number of binding sites across all opsins and transcription factors was performed using a Wilcoxon paired signed-rank test computed in R.

### Comparison of opsin expression and TF binding site profiles

We evaluated the correlation between the transcription factor binding sites in the proximal promoter of each opsin and the expression of each opsin among developing *O. niloticus *fry using Mantel's test of two distance matrices. We generated Euclidean distance matrices of the total number of binding sites for 12 transcription factors within the proximal promoter region of each opsin as well as the percent of total opsin expression from developing *O. niloticus *fry, reported in Carleton et al. [32]. We calculated Mantel's test using the 'mantel.randtest' function from the R package ade4 [111]. Approximate p-values were calculated following 500 randomizations of each matrix. All transcription factor numbers and expression values were standardized prior to clustering. We also expanded this analysis to the entire proximal promoter region after calculating a sequence similarity matrix for the entire proximal promoter using the phylip program dnadist.

### Profiling of microRNAs target sites

We searched the 3'-UTRs of each opsin for binding sites matching the target seed of known miRNAs (miRNA) via the SeedMatch algorithm previously used to identify miRNA targets in cichlid UTRs [72]. This algorithm is similar to the TargetScanS algorithm used in other studies to identify miRNA targets [112]. Briefly, non-redundant fish miRNA targets were obtained from miRBase (http://www.mirbase.org[54]; accessed June 2010) and supplemented with several miRNA target sequences identified in cichlids [72]. We searched each opsin 3'-UTR--defined as the ~500 bp region between the transcription end site and the polyadenylation site (AATAAA)--for sequences matching the seeds of miRNAs from this non-redundant library. In order to account for the high rate of false-positives generated by simply searching for matching seed sites, we aligned the 3'-UTR of each cichlid opsin with those from *G. aculeatus, O. latipes, T. nigrovirdis*, the Japanese pufferfish (*Tetrapdon rubripes*), and *D. rerio *in order to identify sites that were conserved across multiple fish species. For this purpose we defined the first 1 kb of sequence downstream of these latter species' opsins as the 3'-UTR and aligned these to the cichlid sequences with MLagan [113]. To account for errors in the alignment of orthologous 3'-UTRs, we counted as conserved the same miRNA target site found within 50 bp of each other across species. For cichlid opsins that lacked orthologs in the other species, we used the nearest paralog (see Additional file 4).

### Resequencing of putative regulatory sequences in Lake Malawi cichlids

We generated a panel of 18 Lake Malawi cichlids that vary in opsin gene expression. In one individual per species, we sequenced approximately 1 kb of DNA upstream of the translation start site for five opsins and CNE 7, as well as 0.5 kb downstream of the *SWS2B *and *LWS *opsins. We generated primers for these regions based on the *O. niloticus *and *M. zebra *BAC assemblies. The taxa sampled are listed in Additional file 11 along with their GenBank accession numbers; the primers used to generate these sequences are listed in Additional File 12. We measured opsin expression for each individual following the protocols described in Spady et al. [28] and Hofmann et al. [26]. Described briefly, we dissected whole retinas from individual fish and extracted whole RNA from them using Qiagen Qiashredder and RNeasy RNA extraction kits (Valencia, CA). We quantified each RNA sample via spectral absorption, and then reverse transcribed 0.5 μg using Superscript III (Invitrogen). We used previously developed Taqman primers and probes to individually quantify the expression of each opsin in these samples; however, as in our previous studies [26,28], we quantified the expression of the two *RH2A *paralogs jointly. Reaction efficiencies for each opsin were standardized relative to an internal construct developed especially for this purpose and described in Spady et al. [28].

Following re-sequencing of the candidate *cis*-regulatory regions, we estimated polymorphism statistics for the resulting sequences, and also performed a sliding-window analysis of nucleotide diversity (π), in the program DnaSP v5 [114]. For the sliding-window analysis, we ignored all gaps and specified a window length of 50 bp and a step size of 10 bp. Finally, we calculated the statistical association between polymorphisms found in CRX binding sites and other peaks of nucleotide diversity among the sampled taxa using linear regression in the program gPLINK v1.07 [115]. For each test, we estimated the association of each locus with the expression of its downstream opsin under an additive genetic model, using membership in one of two major phylogenetic clades (mbuna and utaka; see Additional file 8) as a covariate.

## Abbreviations

BAC: bacterial artificial chromosome; CDS: protein-coding sequence; CNE: conserved non-coding element; D_xy_: pairwise sequence divergence; HWE: Hardy-Weinberg equilibrium; INT: intronic sequence; LG: linkage group; MAF: minor allele frequency; miRNA: microRNA; P_div_: proportion divergence TFBS/miRNA target sites; PRO: proximal promoter region; QTL: quantitative trait locus; SNP: single nucleotide polymorphism; TFBS: transcription factor binding site; TSS: translation start site; UTR: untranslated region

## Authors' contributions

KEO participated in BAC annotation, carried out the survey of transcription factor binding sites, participated in the sequencing of opsin proximal promoters, participated in the survey of miRNA target sites, performed all statistical analysis, and wrote the manuscript. DS participated in the BAC assembly and annotation. ZN and JS both participated in the sequencing of opsin proximal promoters. SDE sequenced the *LWS *and *SWS2B *3'-UTRs. YHL and JTS performed the search of microRNA target sites. JLB performed the BAC sequencing. KLC designed the study; aided in the BAC screening, sequencing, and assembly; participated in BAC annotation; carried out the analysis of opsin gene expression, and participated in the drafting of the manuscript. All authors read and approved the final manuscript.

## Supplementary Material

Additional file 1 **Synteny (Pip plots) of *O. niloticus *and *M. zebra *opsin-containing BAC sequences**. Click here for file

Additional file 2 **Synteny (Pip plots) of *O. niloticus *opsin-containing BACs against the genome assemblies of five teleost species**. Click here for file

Additional file 3 **Opsin gene content of five teleost genomes**. Phylogeny of the teleost taxa is recreated from [118]. Click here for file

Additional file 4 **Orthology of *RH2 *and *SWS2 *opsin paralogs from five teleost fish genomes**. A) *RH2 *phylogeny. B) *SWS2 *phylogeny. In both cases, broken lines indicate branches leading from the outgroup that were shortened to fit each tree into the figure; these do not represent missing or incomplete branch length information. Click here for file

Additional file 5 **FASTA file of 20 conserved non-coding elements (CNEs), promoter sequences, 3-UTRs, and seven non-opsin promoters from *O. niloticus *and *M. zebra *(80 sequences total)**. Click here for file

Additional file 6 **Complete transcription factor binding site profiles for 20 CNEs in *O. niloticus *and *M. zebra***. Click here for file

Additional file 7 **Complete list of miRNA target sites identified within the 3'-UTR of each opsin in *O. niloticus *and *M. zebra***. Click here for file

Additional file 8 **Names, opsin expression values, and polymorphisms found within the proximal promoters of 18 Lake Malawi cichlid species**. Click here for file

Additional file 9 **Length and D_xy _scores between *O. niloticus *and *M. zebra *for each coding and non-coding region examined**. Click here for file

Additional file 10 **Identification of opsin-containing BACs from Finger Printed Contigs**. A-C) BACs fingerprinted contig containing the *SWS2A*-*SWS2B*-*LWS *(A) *RH2 *(B) and *SWS1 *(C) genes. Arrows indicate PCR products successfully amplified using primers designed to BAC end sequences for clones whose names are shown in the corresponding color. Colored circles are the approximate locates of each gene. Click here for file

Additional file 11 **GenBank accession numbers for all sequences generated in this study**. Click here for file

Additional file 12 **Primers used to amplify and sequence the proximal promoter regions and 3'-UTR of several opsins from 18 Lake Malawi cichlid species**. Click here for file
