# Supplementary figures and images for: Divergence in cis-regulatory sequences surrounding the opsin gene arrays of African cichlid fishes
Source: BMC Evol Biol. 2011 May 9;11:120. doi: 10.1186/1471-2148-11-120 (PMC3116502; doi:10.1186/1471-2148-11-120)

Additional file 1

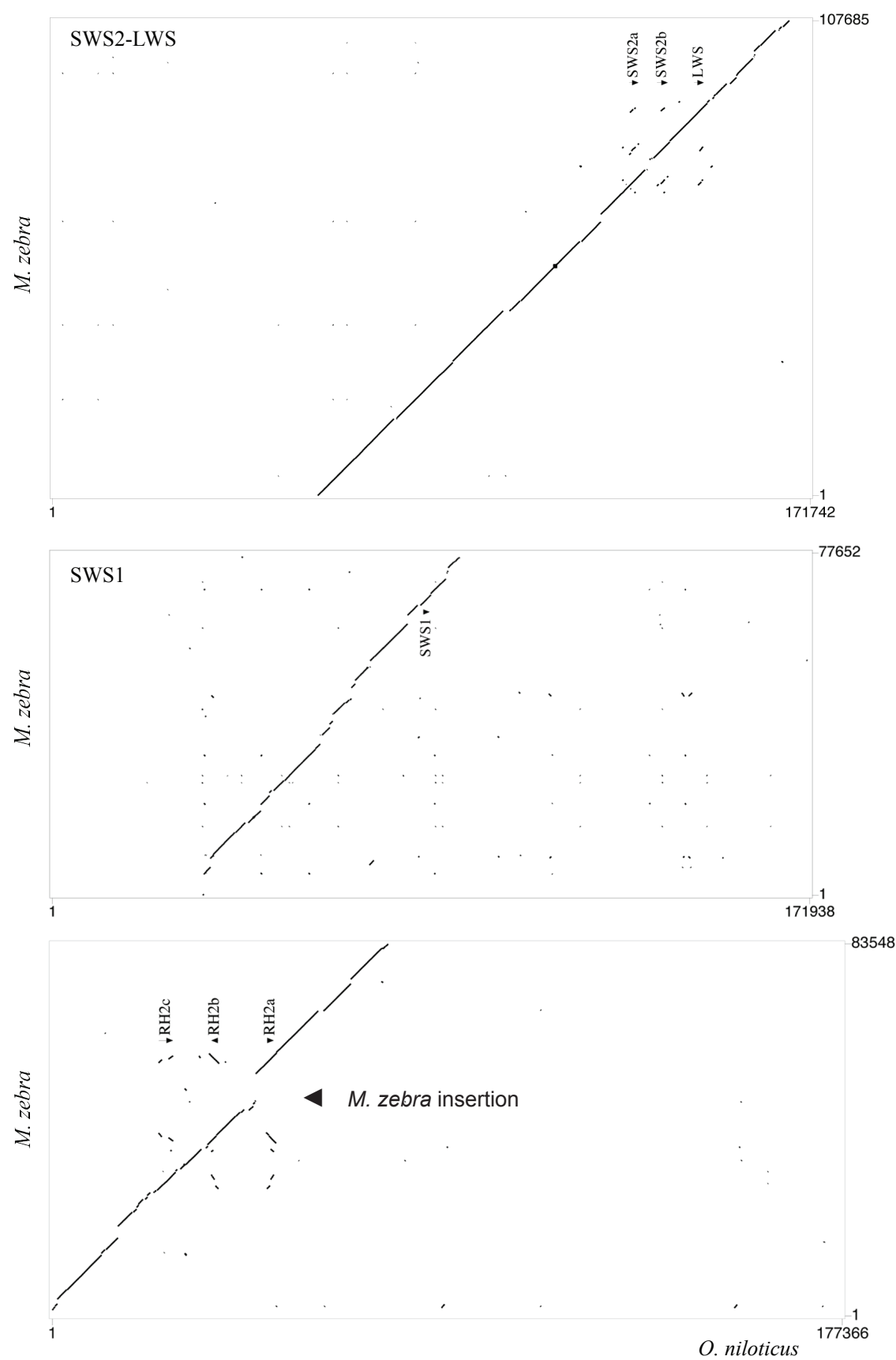

Supplement: Additional file 1 — Synteny (Pip plots) of O. niloticus and M. zebra opsin-containing BAC sequences. [file 1471-2148-11-120-S1.PDF]

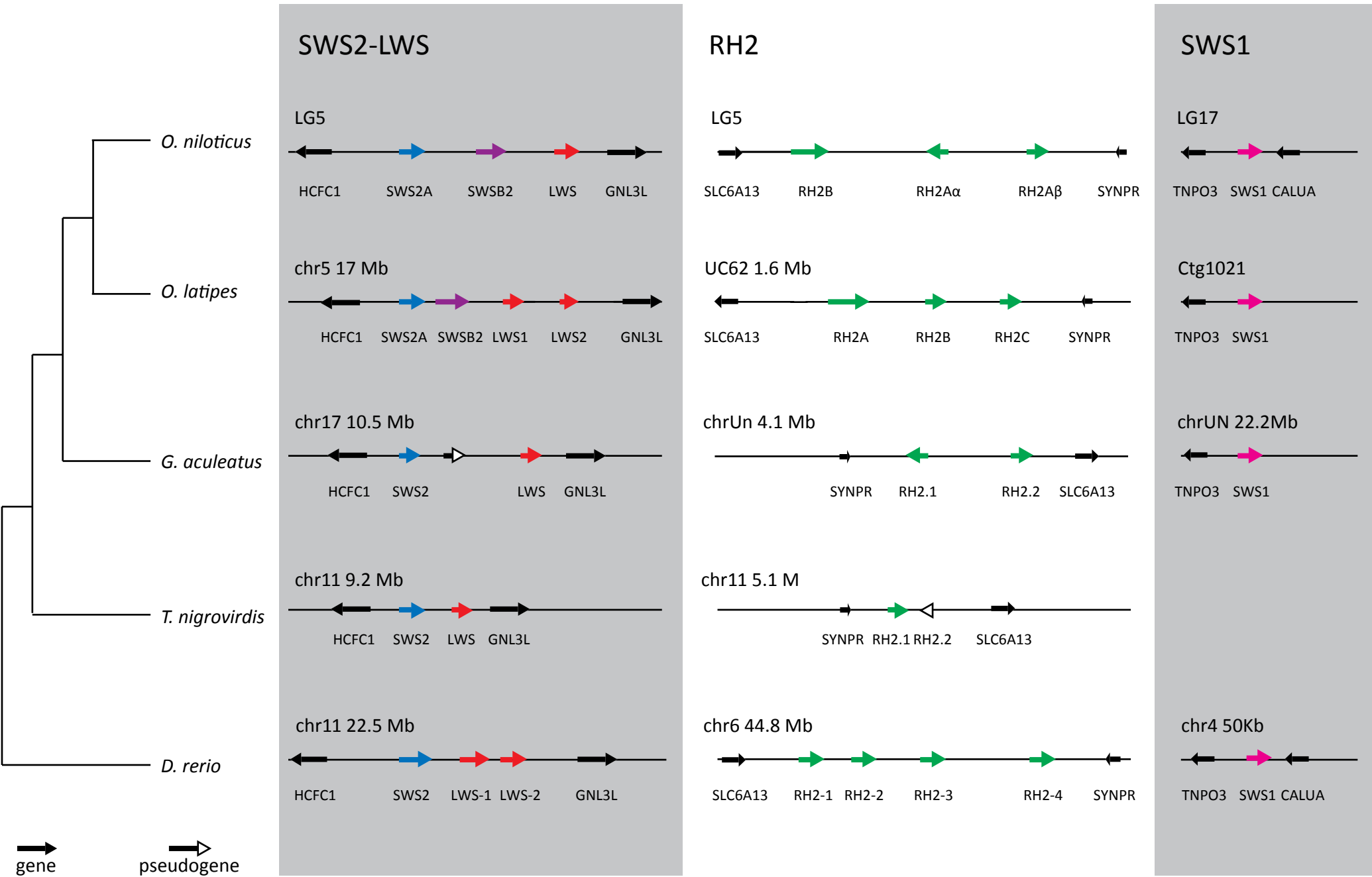

Supplement: Additional file 3 — Opsin gene content of five teleost genomes. Phylogeny of the teleost taxa is recreated from [118]. [file 1471-2148-11-120-S3.PDF]

# Additional file 4

A

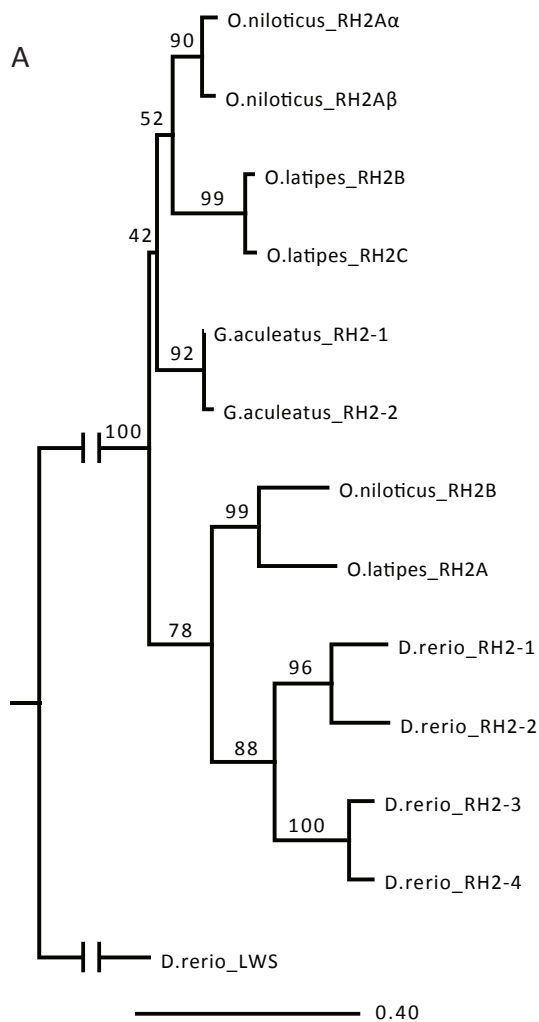

B

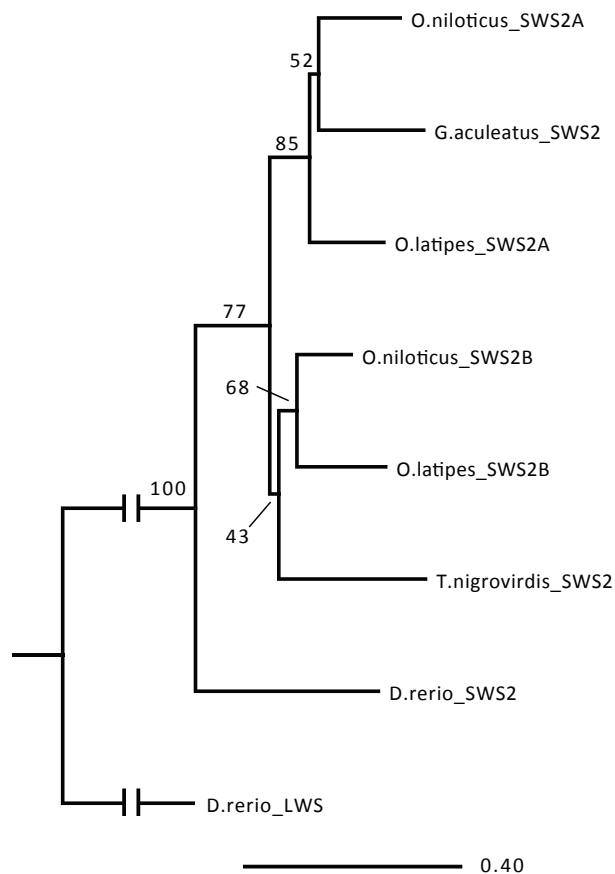

Supplement: Additional file 4 — Orthology of RH2 and SWS2 opsin paralogs from five teleost fish genomes. A) RH2 phylogeny. B) SWS2 phylogeny. In both cases, broken lines indicate branches leading from the outgroup that were shortened to fit each tree into the figure; these do not represent missing or incomplete branch length information. [file 1471-2148-11-120-S4.PDF]

A

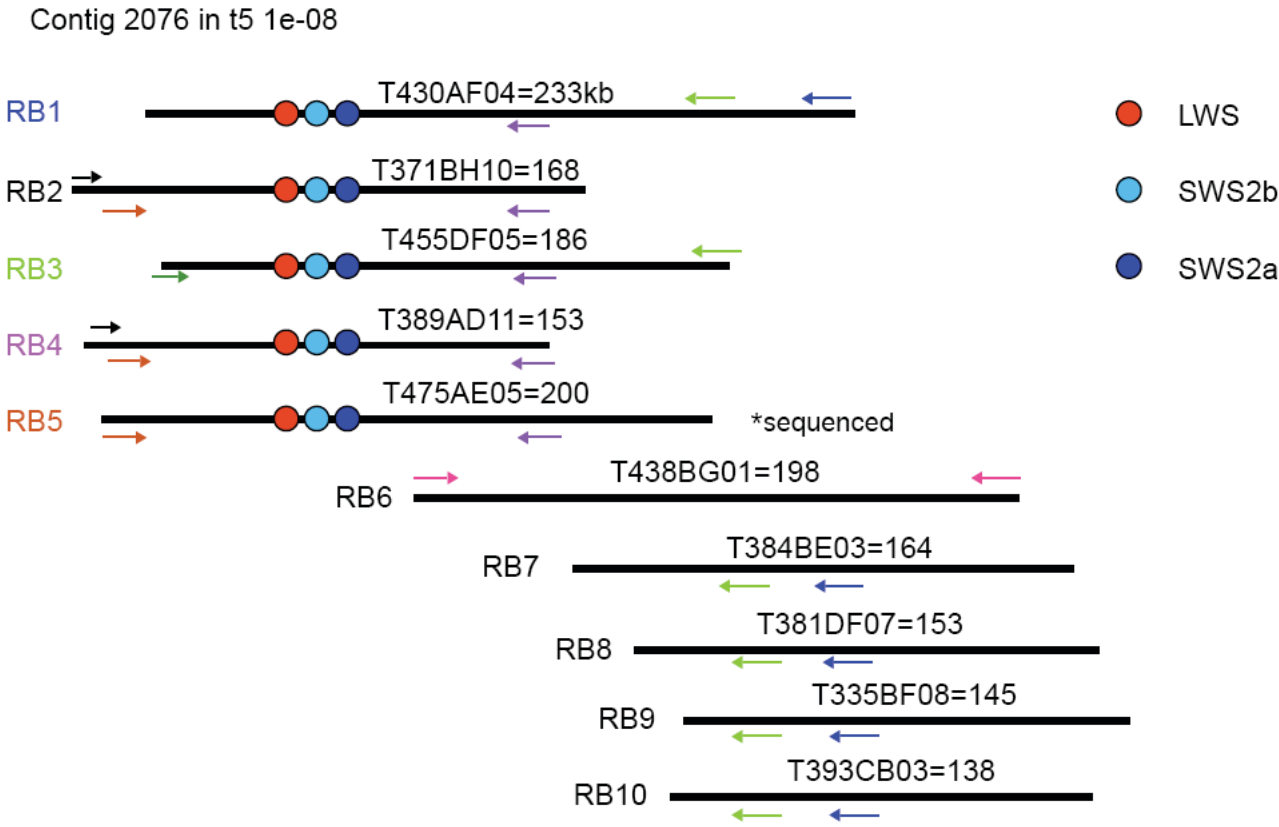

B

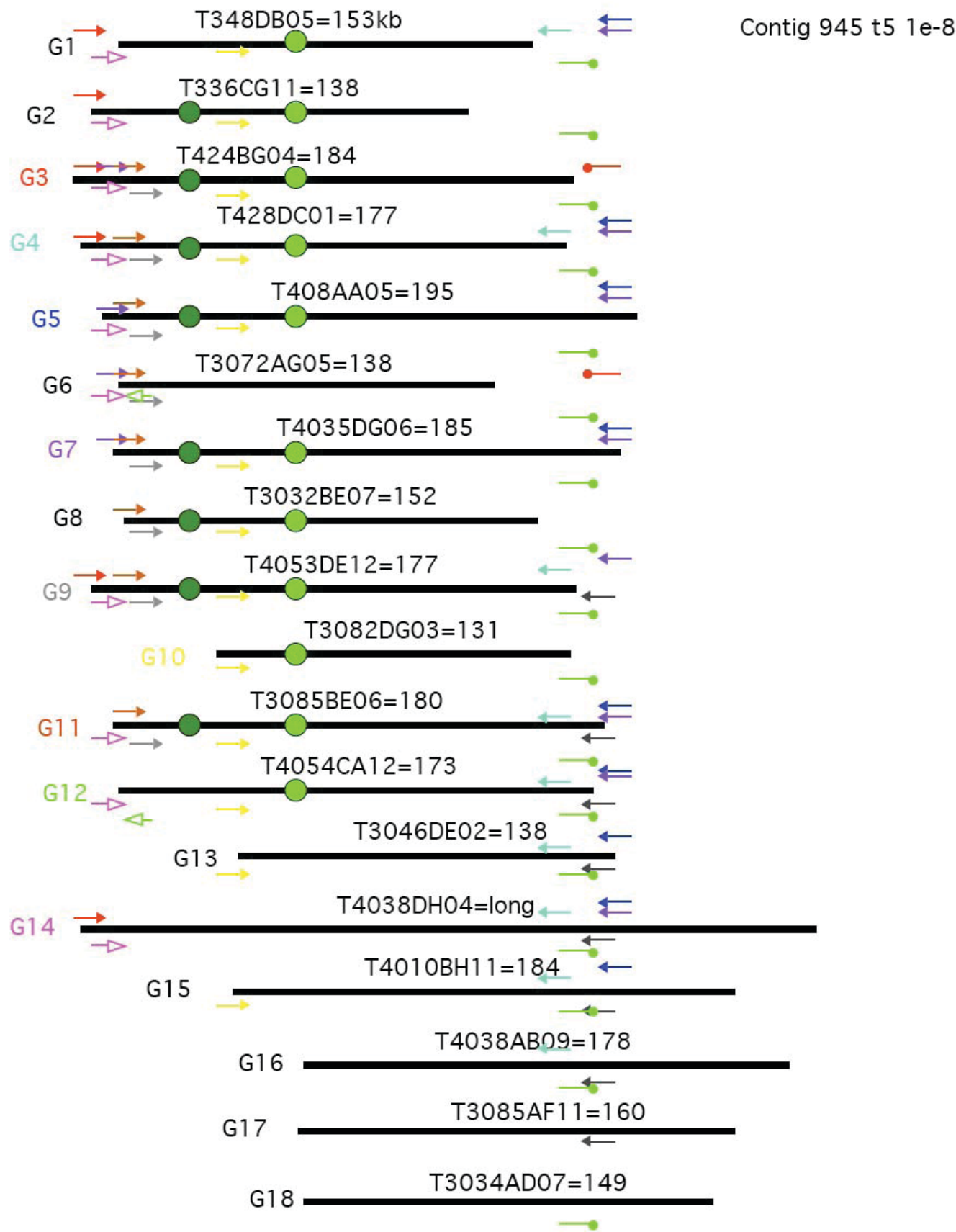

C

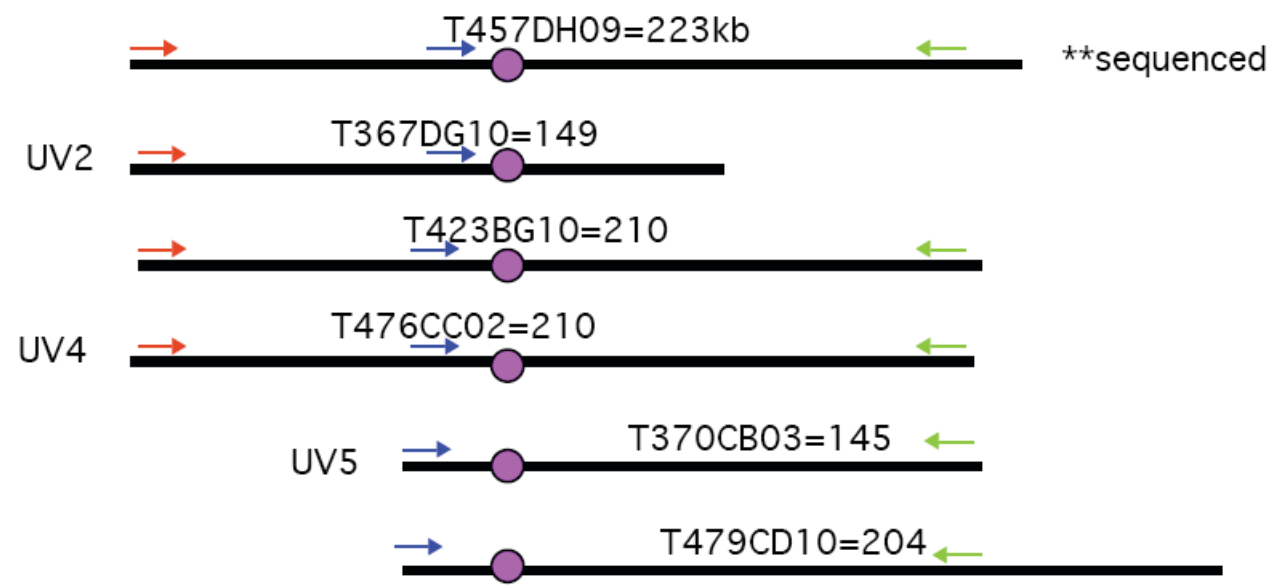

Supplement: Additional file 10 — Identification of opsin-containing BACs from Finger Printed Contigs. A-C) BACs fingerprinted contig containing the SWS2A-SWS2B-LWS (A) RH2 (B) and SWS1 (C) genes. Arrows indicate PCR products successfully amplified using primers designed to BAC end sequences for clones whose names are shown in the corresponding color. Colored circles are the approximate locates of each gene. [file 1471-2148-11-120-S10.PDF]
