# Supplementary material for: Divergence in cis-regulatory sequences surrounding the opsin gene arrays of African cichlid fishes
Source: BMC Evol Biol. 2011 May 9;11:120. doi: 10.1186/1471-2148-11-120 (PMC3116502; doi:10.1186/1471-2148-11-120)
Supplement: Additional file 2 — Synteny (Pip plots) of O. niloticus opsin-containing BACs against the genome assemblies of five teleost species. [file 1471-2148-11-120-S2.PDF]

Additional file 2

A

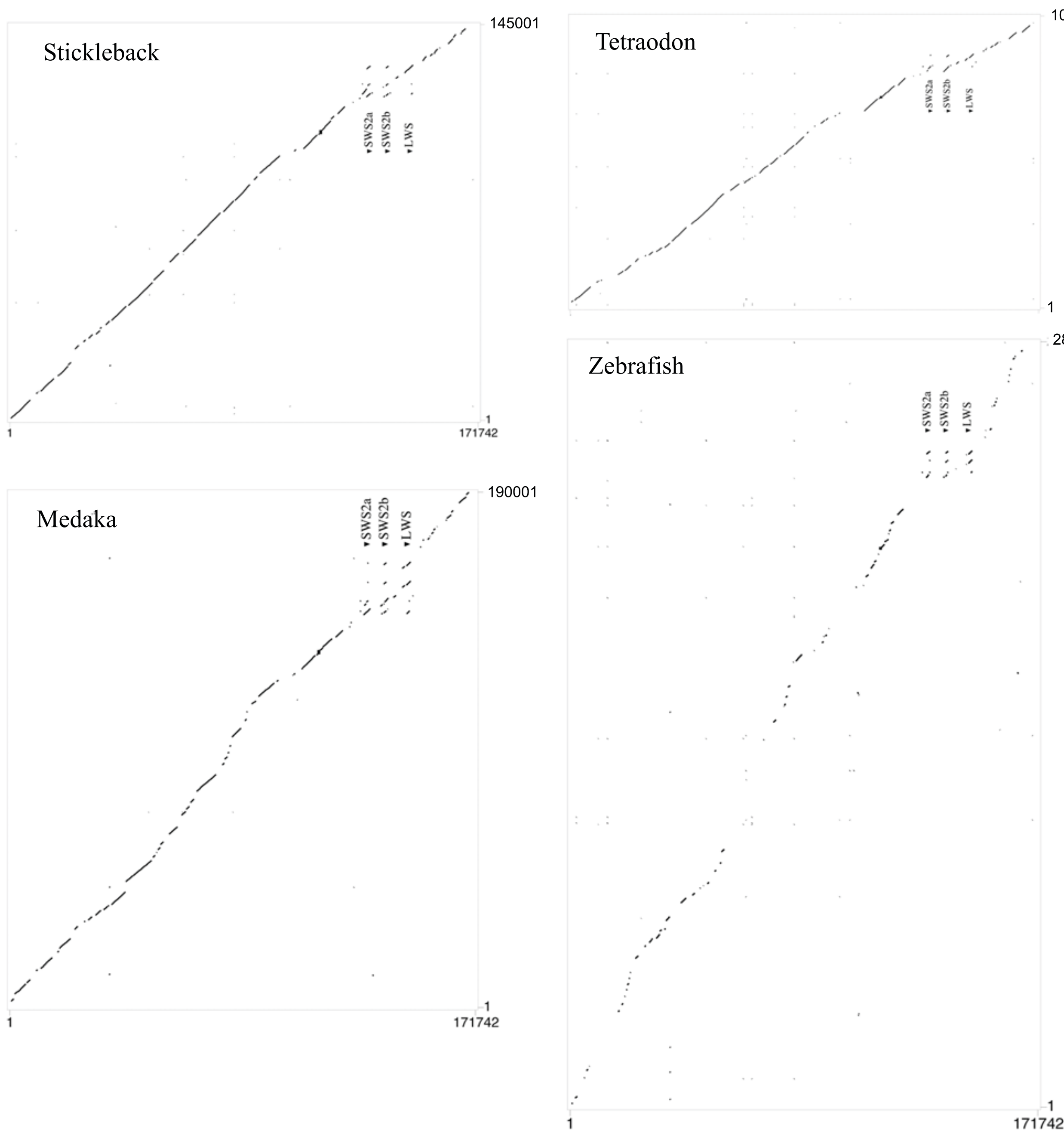

B

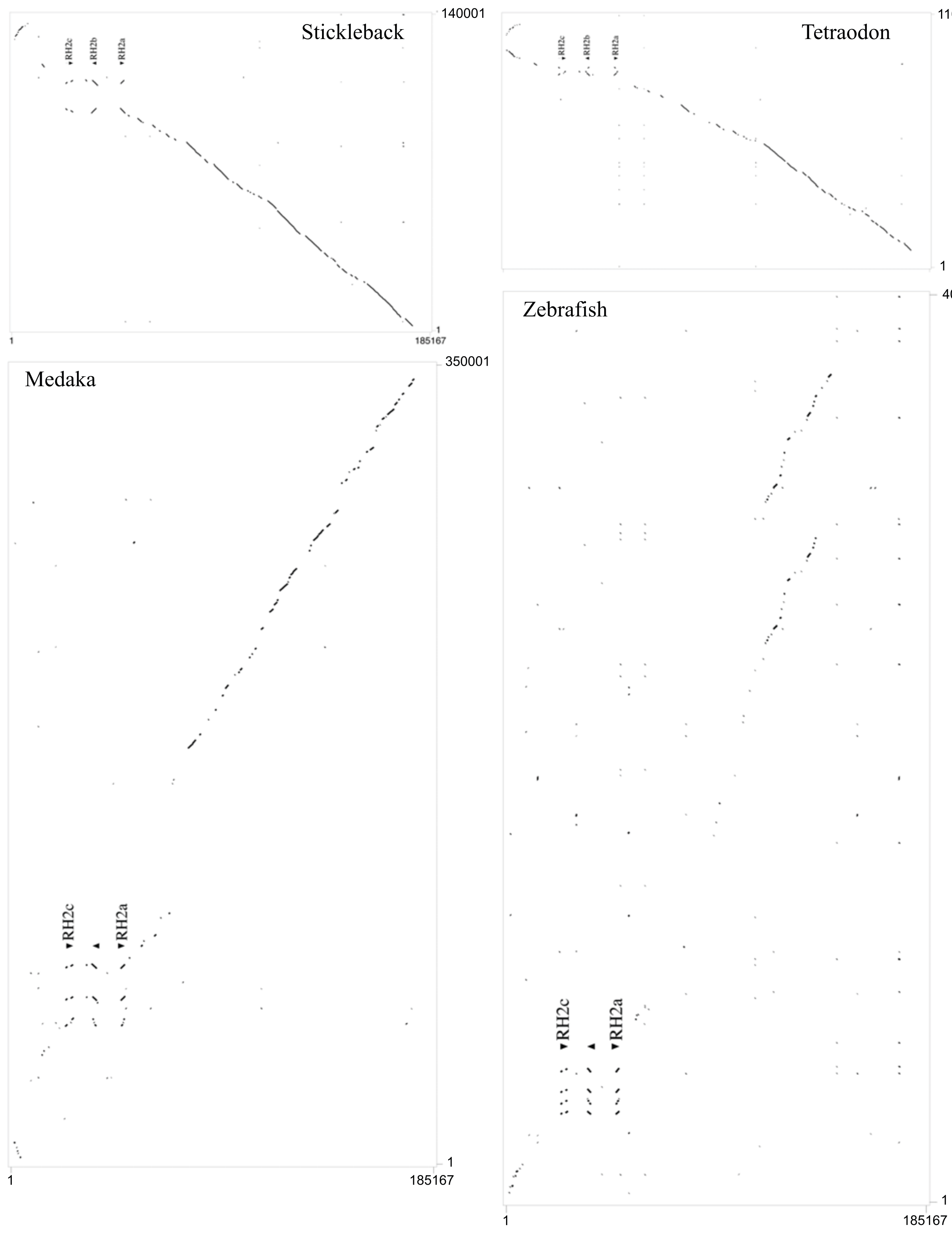

C

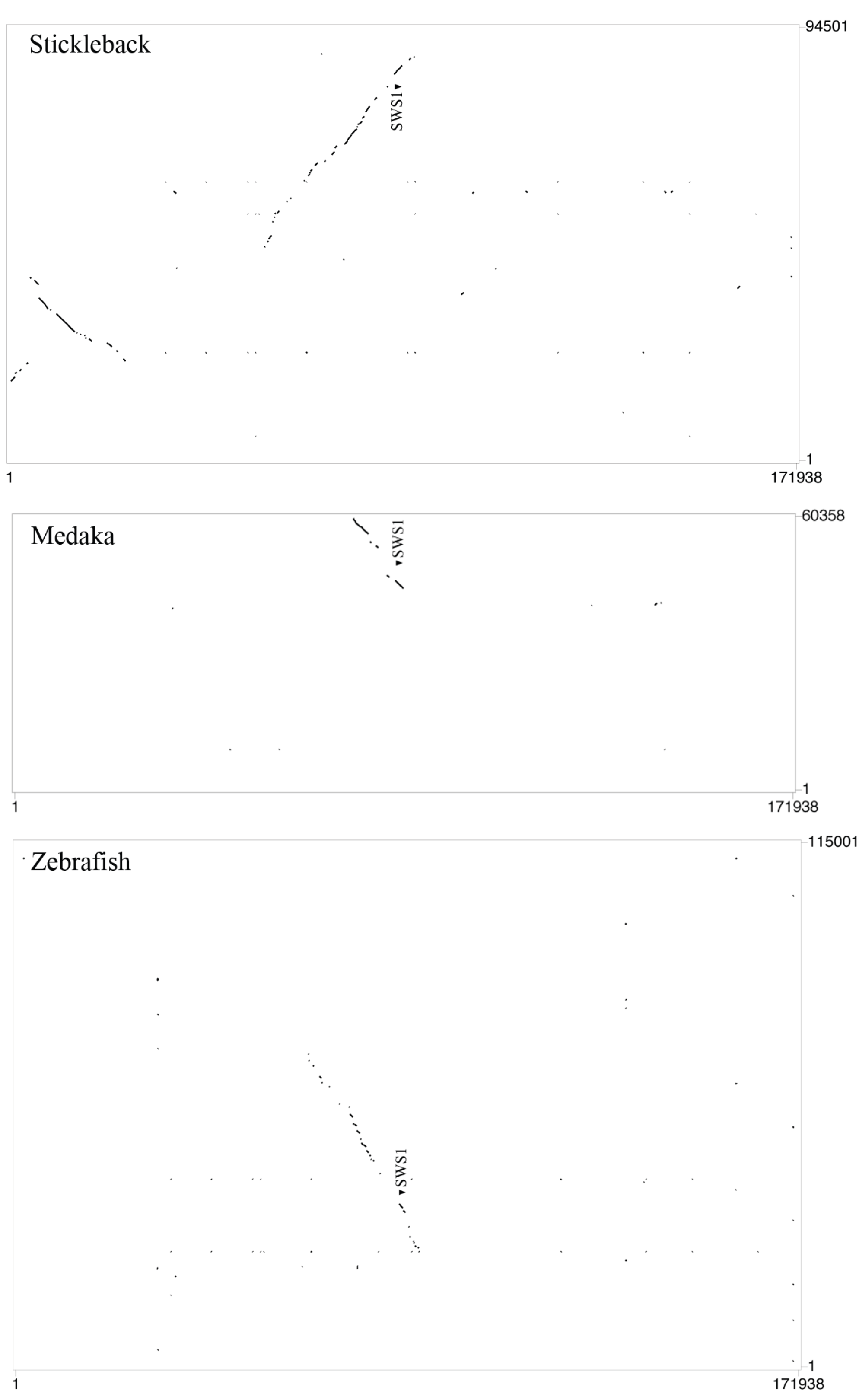

Figure S2C. Synteny between *O. niloticus* SWS1 BAC (171938 bp) and other fishes. The location of the SWS1 gene is shown by the arrow.
